# Integrative modeling of cold and light signaling in tomato: unraveling the transcriptional network governing cold acclimation

**DOI:** 10.48130/els-0026-0007

**Published:** 2026-08-12

**Authors:** Jian-Ping Tao, Ting Huang, Zhi-Hang Hu, Chen Chen, Hui Liu, Xiong You, Ai-Sheng Xiong

**Affiliations:** 1 College of Horticulture, State Key Laboratory of Crop Genetics & Germplasm Enhancement and Utilization, Key Laboratory of Horticultural Crop Biology and Germplasm Creation in East China of the Ministry of Agriculture and Rural Affairs, Nanjing Agricultural University, Nanjing, Jiangsu 210095, China; 2 Institute of Agricultural Information, Jiangsu Academy of Agricultural Sciences, Nanjing, Jiangsu 210014, China; 3 College of Sciences, Nanjing Agricultural University, Nanjing, Jiangsu 210095, China; 4 State Key Laboratory of Tree Genetics and Breeding, Co-Innovation Center for Sustainable Forestry in Southern China, Key Laboratory of Tree Genetics and Biotechnology of Educational Department of China, Key Laboratory of Tree Genetics and Silvicultural Sciences of Jiangsu Province, Nanjing Forestry University, Nanjing, Jiangsu 210037, China

**Keywords:** Light and cold signaling pathway, Cold tolerance, Computational model, *Solanum lycopersicum*, R:FR, Gene regulatory network

## Abstract

The integration of light and cold signaling is critical for cold acclimation in plants, but the regulatory complexity of the underlying transcriptional network remains poorly understood. To systematically dissect this interplay in tomato (*Solanum lycopersicum*), we constructed a computational model that merges the core cold-responsive inducer of CBF expression 1 (ICE1)–C-repeat binding factor (CBF)–cold regulated factors (COR) pathway with light-sensitive modules, including the constitutive photomorphogenic 1 (COP1)–elongated hypocotyl 5 (HY5)–MYB domain protein 15 (MYB15) cascade and the phytochrome–phytochrome interacting factor 4 (PIF4)–GA-INSENSITIVE 4 (GIA4) regulatory axis. Simulations successfully reproduced the experimentally observed gene expression dynamics across different photoperiods and revealed that phytochrome activity is co-modulated by both light quality and temperature. Our model predicts that a low red/far-red ratio enhances the expression of *CBF*, whereas cold treatment stabilizes phyA and phyB proteins, jointly promoting cold tolerance. Additionally, the PIF4–GAI4 negative feedback loop is shown to generate sustained oscillations in *SlPIF4*'s expression, and the cold-responsive gene *SlCOR413* is predicted to exhibit low-temperature-induced oscillatory behavior. This integrative framework provides a systems-level tool for dissecting light–cold crosstalk and offers a basis for rationally engineering cold tolerance in horticultural crops through targeted modulation of transcriptional networks.

## Introduction

Low-temperature stress is a major abiotic factor that limits plants' growth, geographic distribution, and yield, posing a serious threat to global crop production^[1]^. Over the course of evolution, plants have developed sophisticated cold acclimation mechanisms: Upon perceiving cold signals, they activate a cascade of physiological, biochemical, and molecular responses to enhance freezing tolerance^[2]^. Importantly, cold acclimation is not governed by a single signaling pathway but arises from the coordinated integration of multiple environmental and endogenous cues^[3]^. Among these, the crosstalk between light and low-temperature signals plays a particularly pivotal role.

Light supplies energy for photosynthesis and also serves as a critical environmental signal that regulates plants' growth, development, and stress resilience. Photoreceptors, such as phytochromes and cryptochromes, detect the quality, intensity, and duration of light, thereby modulating the activity and expression of downstream transcription factors^[4,5]^. Conversely, low-temperature signaling primarily triggers the expression of cold-responsive genes and promotes the accumulation of protective compounds through well-conserved pathways, including the C-repeat binding factor (CBF) regulatory module^[6,7]^. Growing evidence indicates extensive interactions between the light and cold signaling networks. For example, light signals enhance the efficiency of cold responses by regulating the circadian expression of key CBF pathway components; in turn, cold stress influences the expression and stability of photoreceptors and light-responsive genes^[8]^. This synergistic integration critically determines the effectiveness and amplitude of cold acclimation, representing a central regulatory hub that enables plants to precisely adapt to low temperatures.

Despite progress in understanding how light or cold signals independently contribute to cold tolerance, the transcriptional architecture underlying their integration remains poorly resolved. Most existing studies have focused on individual genes or limited pathway interactions, failing to systematically capture the hierarchy of core transcription factors, the network of gene–gene interactions, and the key signaling nodes that converge during light–cold signals' integration^[9,10]^. Consequently, the dynamic properties and regulatory logic of this integrated network are still elusive, limiting our ability to predict and manipulate cold acclimation in crops.

In this study, we developed a integrative computational model that unifies core light- and cold-responsive transcriptional modules in tomato (*Solanum lycopersicum*). By coupling the core INDUCER OF CBF EXPRESSION 1—C-REPEAT BINDING FACTOR 1—COLD REGULATED FACTORS (ICE1–CBF–COR) cold-signaling axis with light-sensitive cascades, namely, CONSTITUTIVE PHOTOMORPHOGENIC 1—ELONGATED HYPOCOTYL 5—MYB DOMAIN PROTEIN 15 (COP1–HY5–MYB15) and phytochromes—PHYTOCHROME INTERACTING FACTOR 4—GA-INSENSITIVE 4 (phytochromes-PIF4–GIA4), we established a systems-level platform for dissecting the regulatory logic by which light quality and photoperiod govern cold acclimation. Furthermore, this study identifief previously uncharacterized regulatory interactions and furnishes testable, predictive insights for improving cold tolerance via the rational engineering of network hubs.

## Materials and methods

A computational model was constructed to represent the coupled cold and light signaling pathways in tomato. The network integrates three experimentally validated regulatory modules: (1) the core cold response pathway, ICE1–CBF–COR; (2) the light-regulated COP1–HY5–MYB15 module; and (3) the light-quality-sensing phytochromes–PIF4–GAI4 module. These modules are interconnected through shared transcription factors and environmental inputs (light intensity, light quality, and temperature), enabling dynamic crosstalk between photomorphogenesis and cold acclimation. A detailed description of the derivation of the gene regulatory network is provided in the Supplementary Text 1.

### Plant materials and growth conditions

All experimental data used for model parameterization and validation were derived from published studies on tomato. For parameter estimation, we obtained *SlHY5* expression data from wild-type (*Solanum lycopersicum* cv. 'Ailsa Craig') seedlings, and *SlMYB15* expression data from wild-type, *hy5* mutant, and *HY5*-overexpressing lines^[11]^. These plants were grown under a 12-h light/12-h dark photoperiod with a photosynthetic photon flux density of 600 μmol m^−2^ s^−1^ and 85% relative humidity. For cold stress treatments, plants were exposed to 4 °C under the same light conditions. Model validation used *SlCBF1* and *SlCOR413* expression data from wild-type plants grown at 4 °C under either a 16-h light/8-h dark photoperiod or continuous light (LL) following a 16-h light period^[12−14]^. All experimental procedures and data collection followed the protocols described in the original publications.

### Temperature signal transduction modelling

According to the temperature-sensing model^[15,16]^, calcium enters the cytoplasm through channels whose activity is a function of the cooling rate $ \left(\dfrac{\mathrm{d}T}{\mathrm{d}t}\right) $, and it is removed by pumps whose activity varies with absolute temperature (*T*). The related equations are as follows:

1 $ \dfrac{\mathrm{d}Iexmax}{\mathrm{d}t}={P}_{0}\mathrm{sgn}\left([\mathrm{C}{\mathrm{a}}^{2+}]-{\left[\mathrm{C}{\mathrm{a}}^{2+}\right]}_{0}\right)\dfrac{[\mathrm{C}{\mathrm{a}}^{2+}]}{{K}_{P}+[\mathrm{C}{\mathrm{a}}^{2+}]} , $

where, $ \mathrm{sgn(}x) $ is signal function, and $ {\left[\mathrm{C}{\mathrm{a}}^{2+}\right]}_{0} $ is always set to 0.1 nM. The free parameters $ {P}_{0} $ and $ {K}_{P} $ were set as 0.005 μM s^−2^ and 0.5 μM s^−1^, respectively.

2 $ \dfrac{\mathrm{d}[\mathrm{C}{\mathrm{a}}^{2+}]}{\mathrm{d}t}=Ii{n}_{0}+Iinmax\dfrac{{\left| \mathrm{d}T\right| }^{3}}{KK_{1}^{3}+{\left| \mathrm{d}T\right| }^{3}}-Iexmax{\mathrm{e}}^{{{K}_{Q}}(T-{{T}_{0}})}\dfrac{[\mathrm{C}{\mathrm{a}}^{2+}]}{KK_{m}^{2}+\left[\mathrm{C}{\mathrm{a}}^{2+}\right]} , $

where, $ {K}_{Q}=\dfrac{\log Q}{10} $, * Q=10 *, $ K{K}_{1}=1 $ nM, $ K{K}_{m}=0.5 $ nM, $ Ii{n}_{0}=0.005 $ nM s^−1^, and * Iinmax=0.108 * nM s^−1^.

Following cold treatment, calmodulin (CaM) is activated by binding with Ca^2^^+^. This activation follows a cooperative binding curve described by a Hill function with a coefficient of 4. The fraction of activated calmodulin is therefore given by Eq. (3):

3 $ \dfrac{\mathrm{d}CaM}{\mathrm{d}t}=\dfrac{{\left[\mathrm{C}{\mathrm{a}}^{2+}\right]}^{4}}{{K}_{Ca}+{\left[\mathrm{C}{\mathrm{a}}^{2+}\right]}^{4}} . $

### Downstream cold-light response modeling

The model consists of ordinary differential equations (ODEs) listed as Eqs (4)−(24). A description of each of the variables and parameters can be found in Supplementary Tables S1 and S2. The time evolution of the mRNA and protein levels are governed by the following differential equations.

(a) Inhibitor SlCOP1 and activator SlHY5

4 $ \dfrac{\mathrm{d[SlCOP1]}}{\mathrm{d}t}=({p}_{0}+{p}_{0D}D)-({d}_{0}+{d}_{0L}L\mathrm{)[SlCOP1]} , $

5 $ \dfrac{\mathrm{d[MSlHY5]}}{\mathrm{d}t}={v}_{1}\dfrac{1}{1+{\left(\dfrac{\left[\mathrm{SlCOP1}\right]}{{K}_{1}}\right)}^{{{n}_{1}}}}-{k}_{1}\mathrm{[MSlHY5]} , $

6 $ \dfrac{\mathrm{d[SlHY5]}}{\mathrm{d}t}={p}_{1}\left[\mathrm{MSlHY5}\right]-({d}_{1}-{d}_{1L}L+{d}_{1D}D\mathrm{)[SlHY5]} , $

(b) Positive transcription factors (TF): *SlMYB15*, *SlICE1*, and *SlCAMTA* and the negative TF *SlZAT12*

7 $ \dfrac{\mathrm{d[MSlMYB15]}}{\mathrm{d}t}={v}_{2}\dfrac{{\left(\dfrac{\left[\mathrm{SlHY5}\right]}{{K}_{2}}\right)}^{{{n}_{2}}}}{1+{\left(\dfrac{\left[\mathrm{SlHY5}\right]}{{K}_{2}}\right)}^{{{n}_{2}}}}-{k}_{2}\mathrm{[MSlMYB15]} , $

8 \begin{equation*}\begin{split} \dfrac{\mathrm{d[SlMYB15]}}{\mathrm{d}t}=\;&{p}_{2}\left[\mathrm{MSlMYB15}\right]-{v}_{\mathrm{P1}}\left(1-\dfrac{1}{1+\dfrac{\left[\mathrm{SlMYB15}\right]}{{K}_{\mathrm{P1}}}+\dfrac{CaM}{{K}_{CaM1}}}\right)+\\&{v}_{\mathrm{P2}}\dfrac{\left[\mathrm{SlMYB15P}\right]}{{K}_{\mathrm{P2}}+\left[\mathrm{SlMYB15P}\right]}-{d}_{2}\mathrm{[SlMYB15]} , \end{split}\end{equation*}

9 \begin{equation*}\begin{split}\dfrac{\mathrm{d[SlMYB15P]}}{\mathrm{d}t}=\;&{v}_{\mathrm{P1}}\left(1-\dfrac{1}{1+\dfrac{\left[\mathrm{SlMYB15}\right]}{{K}_{\mathrm{P1}}}+\dfrac{CaM}{{K}_{CaM1}}}\right)-\\&{v}_{\mathrm{P2}}\dfrac{\left[\mathrm{SlMYB15P}\right]}{{K}_{\mathrm{P2}}+\left[\mathrm{SlMYB15P}\right]}-{d}_{P2}\mathrm{[SlMYB15P]} ,\end{split}\end{equation*}

10 \begin{equation*}\begin{split}\dfrac{\mathrm{d}\left[\mathrm{SlICE1}\right]}{\mathrm{d}t}=\;&p-{v}_{\mathrm{P3}}\left(1-\dfrac{1}{1+\dfrac{\left[\mathrm{SlICE1}\right]}{{K}_{\mathrm{P3}}}+\dfrac{CaM}{{K}_{CaM2}}}\right)+\\&{v}_{\mathrm{P4}}\dfrac{\left[\mathrm{SlICE1P}\right]}{{K}_{\mathrm{P4}}+\left[\mathrm{SlICE1P}\right]}-d\mathrm{[SlICE1]} , \end{split}\end{equation*}

11 \begin{equation*}\begin{split}\dfrac{\mathrm{d}\left[\mathrm{SlICE1P}\right]}{\mathrm{d}t}=\;&{v}_{\mathrm{P3}}\left(1-\dfrac{1}{1+\dfrac{\left[\mathrm{SlICE1}\right]}{{K}_{\mathrm{P3}}}+\dfrac{CaM}{{K}_{CaM2}}}\right)-\\&{v}_{\mathrm{P4}}\dfrac{\left[\mathrm{SlICE1P}\right]}{{K}_{\mathrm{P4}}+\left[\mathrm{SlICE1P}\right]}-{d}_{P3}\mathrm{[SlICE1P]} , \end{split}\end{equation*}

12 $ \dfrac{\mathrm{d}\left[\mathrm{MSlCAMTA}\right]}{\mathrm{d}t}={v}_{3}\dfrac{{\left(\dfrac{CaM}{{K}_{CaM3}}\right)}^{{{n}_{3}}}}{1+{\left(\dfrac{CaM}{{K}_{CaM3}}\right)}^{{{n}_{3}}}}-{k}_{3}\left[\mathrm{MSlCAMTA}\right] , $

13 $ \dfrac{\mathrm{d}\left[\mathrm{SlCAMTA}\right]}{\mathrm{d}t}={p}_{3}\left[\mathrm{MSlCAMTA}\right]-{d}_{3}\left[\mathrm{SlCAMTA}\right] , $

(c) Output: *SlCBF1* mRNA, and phosphorylated and nonphosphorylated SlCBF1 protein

14 \begin{equation*}\begin{split}& \dfrac{\mathrm{d}\left[\mathrm{MSlCBF1}\right]}{\mathrm{d}t}={v}_{4}\dfrac{{\left(\dfrac{\left[\mathrm{SlICE1}\right]}{{K}_{3}}\right)}^{{{n}_{5}}}}{1+{\left(\dfrac{\left[\mathrm{SlICE1}\right]}{{K}_{3}}\right)}^{{{n}_{5}}}}\\&\left( 1+\dfrac{1}{1 +{\left(\dfrac{\left[\mathrm{SlMYB15}\right]}{{K}_{4}}\right)}^{{{n}_{6}}} +{\left(\dfrac{\left[\mathrm{SlHY5}\right]}{{K}_{5}}\right)}^{{{n}_{7}}} +{\left(\dfrac{\left[\mathrm{SlPIF4}\right]}{{K}_{6}}\right)}^{{{n}_{8}}} +{\left(\dfrac{\left[\mathrm{SlCAMTA}\right]}{{K}_{7}}\right)}^{{{n}_{9}}}} \right)\\& \dfrac{1}{1+{\left(\dfrac{\left[\mathrm{ZAT12}\right]}{{K}_{8}}\right)}^{{{n}_{10}}}}-{k}_{4}\left[\mathrm{MSlCBF1}\right] , \end{split}\end{equation*}

15 \begin{equation*}\begin{split}\dfrac{\mathrm{d}\left[\mathrm{SlCBF1}\right]}{\mathrm{d}t}=\;&{p}_{4}\left[\mathrm{MSlCBF1}\right]-{v}_{\mathrm{P5}}\dfrac{\dfrac{\left[SlCBF1\right]}{{K}_{P5}}+\dfrac{CaM}{{K}_{CaM5}}}{1+\dfrac{\left[SlCBF1\right]}{{K}_{P5}}+\dfrac{CaM}{{K}_{CaM5}}}+\\&{v}_{\mathrm{P6}}\dfrac{\left[\mathrm{SlCBF1P}\right]}{{K}_{\mathrm{P6}}+\left[\mathrm{SlCBFP}\right]}-{d}_{4}\mathrm{[SlCBF1]} ,\end{split}\end{equation*}

16 \begin{equation*}\begin{split}\dfrac{\mathrm{d}\left[\mathrm{SlCBF1P}\right]}{\mathrm{d}t}=\;&{v}_{\mathrm{P5}}\left(1-\dfrac{1}{1+\dfrac{\left[\mathrm{SlCBF1}\right]}{{K}_{\mathrm{P5}}}+\dfrac{CaM}{{K}_{CaM5}}}\right)-\\&{v}_{\mathrm{P6}}\dfrac{\left[\mathrm{SlCBF1P}\right]}{{K}_{\mathrm{P6}}+\left[\mathrm{SlCBF1P}\right]}-{d}_{\mathrm{P4}}\left[\mathrm{SlCBF1P}\right] , \end{split}\end{equation*}

(d) Downstream of SlCBF1 and output: *SlZAT12* mRNA and protein, and *SlCOR413* mRNA and protein

17 $ \dfrac{\mathrm{d[MSlZAT12]}}{\mathrm{d}t}={v}_{5a}+{v}_{5}\dfrac{{\left(\dfrac{\left[\mathrm{SlCBF1}\right]}{{K}_{9}}\right)}^{{{n}_{11}}}}{1+{\left(\dfrac{\left[\mathrm{SlCBF1}\right]}{{K}_{9}}\right)}^{{{n}_{11}}}}-{k}_{5}\mathrm{[MSlZAT12]} , $

18 $ \dfrac{\mathrm{d[SlZAT12]}}{\mathrm{d}t}={p}_{5}\mathrm{[MSlZAT12]-}{d}_{5}\left[\mathrm{SlZAT12}\right] , $

19 \begin{equation*}\begin{split}\dfrac{\mathrm{d[MSlCOR413]}}{\mathrm{d}t}=\;&{v}_{6}\left(1-\dfrac{1}{1+{\left(\dfrac{\left[\mathrm{SlCBF1}\right]}{{K}_{10}}\right)}^{{{n}_{12}}}+{\left(\dfrac{\left[\mathrm{SlZAT12}\right]}{{K}_{11}}\right)}^{{{n}_{13}}}}\right)\\&-{k}_{6}\left[\mathrm{MSlCOR413}\right] , \end{split}\end{equation*}

20 $ \dfrac{\mathrm{d[SlCOR413]}}{\mathrm{d}t}={p}_{6}\cdot\left[\mathrm{MSlCOR413}\right]-{d}_{6}\left[\mathrm{SlCOR413}\right] , $

(e) The light-quality-induced proteins phyA and phyB and their expression

21 \begin{equation*}\begin{split} \dfrac{\mathrm{d}\left[\mathrm{PHYA}\right]}{\mathrm{d}t}=&{p}_{A}\dfrac{CaM}{{K}_{A}+CaM}+{p}_{BA}\left(1-\dfrac{{\mathrm{[PHYB]}}^{{{n}_{14}}}}{K_{BA}^{{n}_{4}}+{\mathrm{[PHYB]}}^{{{n}_{14}}}}\right)-\\&\dfrac{{d}_{A}}{1+{a}_{FR}\cdot\gamma }\left[\mathrm{PHYA}\right] , \end{split}\end{equation*}

22 $ \dfrac{\mathrm{d}\left[\mathrm{PHYB}\right]}{\mathrm{d}t}={p}_{B}\dfrac{{K}_{B}}{{K}_{B}+CaM}\left(1-\dfrac{{\mathrm{[PHYA]}}^{{{n}_{15}}}}{K_{AB}^{{n}_{5}}+{\mathrm{[PHYA]}}^{{{n}_{15}}}}\right)-\dfrac{{d}_{B}}{1+{a}_{R}\cdot\gamma }\left[\mathrm{PHYB}\right] , $

(f) Negative feedback loop exerted by SlGAI4 on SlPIF4

23 \begin{equation*}\begin{split}\dfrac{\mathrm{d}\left[\mathrm{SlPIF4}\right]}{\mathrm{d}t}=\;&{p}_{PIF}\dfrac{K_{p\mathrm{h}yB}^{{n}_{16}}}{K_{p\mathrm{h}yB}^{{n}_{16}}+{\mathrm{[PHYB]}}^{{{n}_{16}}}}\dfrac{{\mathrm{[PHYA]}}^{{{n}_{17}}}}{{{K_{p\mathrm{h}yA}^{{n}_{17}}}\mathrm{+[PHYA]}}^{{{n}_{17}}}}\\&\dfrac{K_{GAI}^{{n}_{18}}}{K_{GAI}^{{n}_{18}}+{\mathrm{[MSlGAI4]}}^{{{n}_{18}}}}-{d}_{PIF}\left[\mathrm{SlPIF4}\right] , \end{split}\end{equation*}

24 $ \dfrac{\mathrm{d}\left[\mathrm{SlGAI4}\right]}{\mathrm{d}t}={p}_{GAI}\dfrac{{\mathrm{[SlPIF4]}}^{{{n}_{19}}}}{{{K_{PIF}^{{n}_{19}}}\mathrm{+[SlPIF4]}}^{{{n}_{19}}}}-{d}_{GAI}\left[\mathrm{SlGAI4}\right] . $

In the model, * [· ] * denotes the concentrations of mRNAs and their corresponding proteins. The transcription rate is represented by *v*, and *p* denotes either the translation rate or the rate of protein activation. The degradation rate of mRNA is given by *k*, and *d* represents the protein degradation rate. The Michaelis–Menten constant is denoted by *K*. To simplify the model, the Hill coefficients *n_i_* (*i* = 1, ..., 19) in the equations are set to 2, corresponding to the dimeric form of transcriptional regulatory proteins. To assess the impact of the assumed Hill coefficient values, we carried out one-at-a-time sensitivity analyses in which each coefficient (*n*_1_−*n*_19_) was varied individually while the remaining parameters were fixed at their basal levels. The responses of the model were evaluated by tracking the peak amplitude, phase, and half-peak width of *SlCBF1*'s mRNA expression. These analyses confirmed that over the examined range, the model's dynamics were qualitatively robust and the essential kinetic patterns remained largely unchanged.

### Numerical simulation and statistical analysis

All computational and statistical analyses were performed in Python 3.10.4. The system of ODEs was numerically solved using the 'odeint' package. The period and phase were determined according to the methodology described in the Supplementary Text 2.

To evaluate the robustness of the cold response pathway model, we conducted a single-parameter sensitivity analysis (Supplementary Fig. S1). This analysis varied 10 parameters independently over a logarithmic range of 10^−3^ to 10^3^ times their baseline values while keeping all other parameters fixed. The parameters that exhibited obvious differences in magnitude from H2025 were selected.

To quantitatively evaluate the agreement between simulated and experimental expression data, we calculated the coefficient of determination (*R*^2^) and the root mean square error (RMSE) for each gene in both the training (*SlHY5, SlMYB15*) and validation (*SlCBF1, SlCOR413*) datasets (Supplementary Table S3). For each gene, paired simulated and experimental values at identical time points were imported into OriginPro software (OriginLab Corporation, Northampton, MA, USA). The *R*^2^ value was obtained using the built-in linear regression analysis, which measures the proportion of variance in the experimental data explained by the simulations. The RMSE was computed as the square root of the average squared difference between paired simulated and experimental values, using the descriptive statistics or custom calculation function available in OriginPro. All metrics were directly extracted from the software's output.

## Results

### Conserved and divergent regulatory structures in *Arabidopsis thaliana* and tomato cold-acclimation models

Calcium (Ca^2^^+^) functions as a ubiquitous secondary messenger in plants, participating in diverse physiological processes including embryogenesis, immune responses, and fruit development^[17−20]^. Over the past two decades, research has extensively characterized Ca^2^^+^-responsive proteins and their downstream targets, elucidating their roles in plant adaptation to biotic and abiotic stresses, including cold. Upon cold shock, a transient influx of Ca^2^^+^ into the cytosol rapidly elevates cytoplasmic Ca^2^^+^ levels. This signal is perceived by calmodulin (CaM), a key Ca^2^^+^ sensor, which, in turn, activates a kinase cascade to induce the downstream genes essential for cold acclimation^[21,22]^. To maintain the model's simplicity, we retained the one-compartment Ca^2^^+^ formulations from the *Arabidopsis thaliana* cold-acclimation model (referred to as H2025^[23]^), enabling a comparative analysis of the system's dynamics under Ca^2^^+^ pulse. The temperature response in the model (Eqs [1]–[3]) is formulated on the bassis of established physiological mechanisms: Calcium influx into the cytoplasm occurs through calcium-permeable channels whose activity is modeled as a function of the cooling rate $ \left(\dfrac{\mathrm{d}T}{\mathrm{d}t}\right) $, whereas calcium efflux is mediated by calcium pumps whose activity depends on the absolute temperature (*T*).

**Figure 1:**
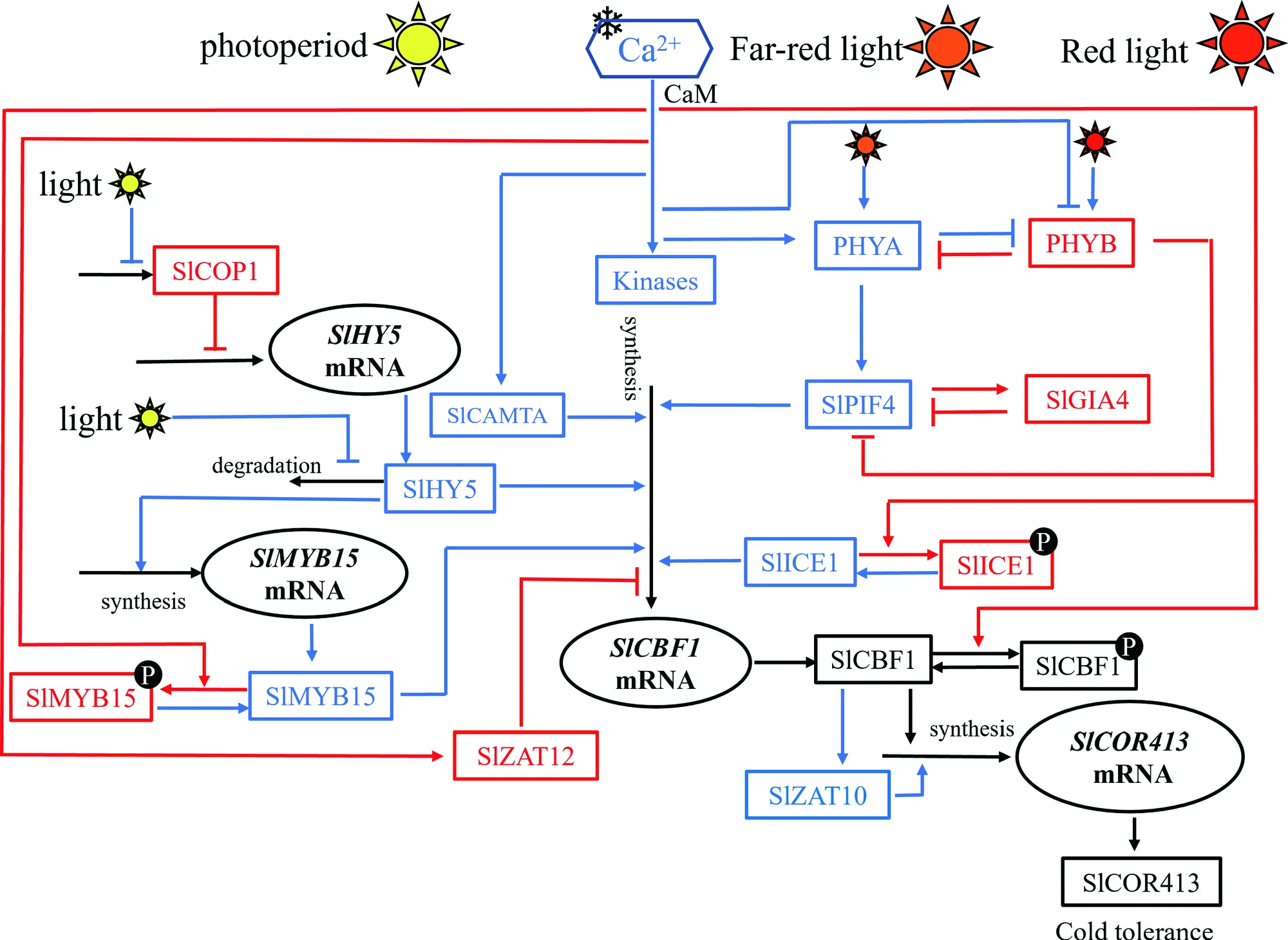
Schematic model for light- and cold-responsive signaling transduction pathways in tomato. Signaling pathway were constructed only in light–dark cycles and red–far red (R:FR) light conditions. This pathway involves negative and positive regulators of the core genes—*SlICE1*, *SlCBF1*, and *SlCOR413*—which are linked with light-related genes and proteins. In order to clarify the regulatory relations in the network, the structure is artificially divided into four modules. The first module is COP1–HY5–MYB15, where HY5 acts as a light-influenced hub. Considering the role of light quality in the pathway, PHY–PIF4–GIA4 is proposed in the photochrome-dependent pathway. Upstream regulators (*SlHY5*, *SlMYB15*, *SlPIF4*, and *SlCAMTA *as activators and *SlZAT12 *as inhibitor) of *CBF* ultimately determine the expression profile of the cold-responsive components in the core ICE–CBF–COR module.

Figure 1 presents the principal scheme of the tomato cold-acclimation model incorporating light signaling. This model was structurally compared with the previously established H2025 model, highlighting newly integrated components and circuit architectures specific to tomato. As in the H2025 framework, the ICE1–CBF–COR pathway remains central to cold stress defense. The tomato model is formalized as a system of 21 variables (Supplementary Table S1) with the corresponding ordinary differential equations (Eqs [4]–[24]) and 80 parameters, which were optimized against the experimental time-series datasets via cost function minimization (see Supplementary Text 3).

**Figure 2:**
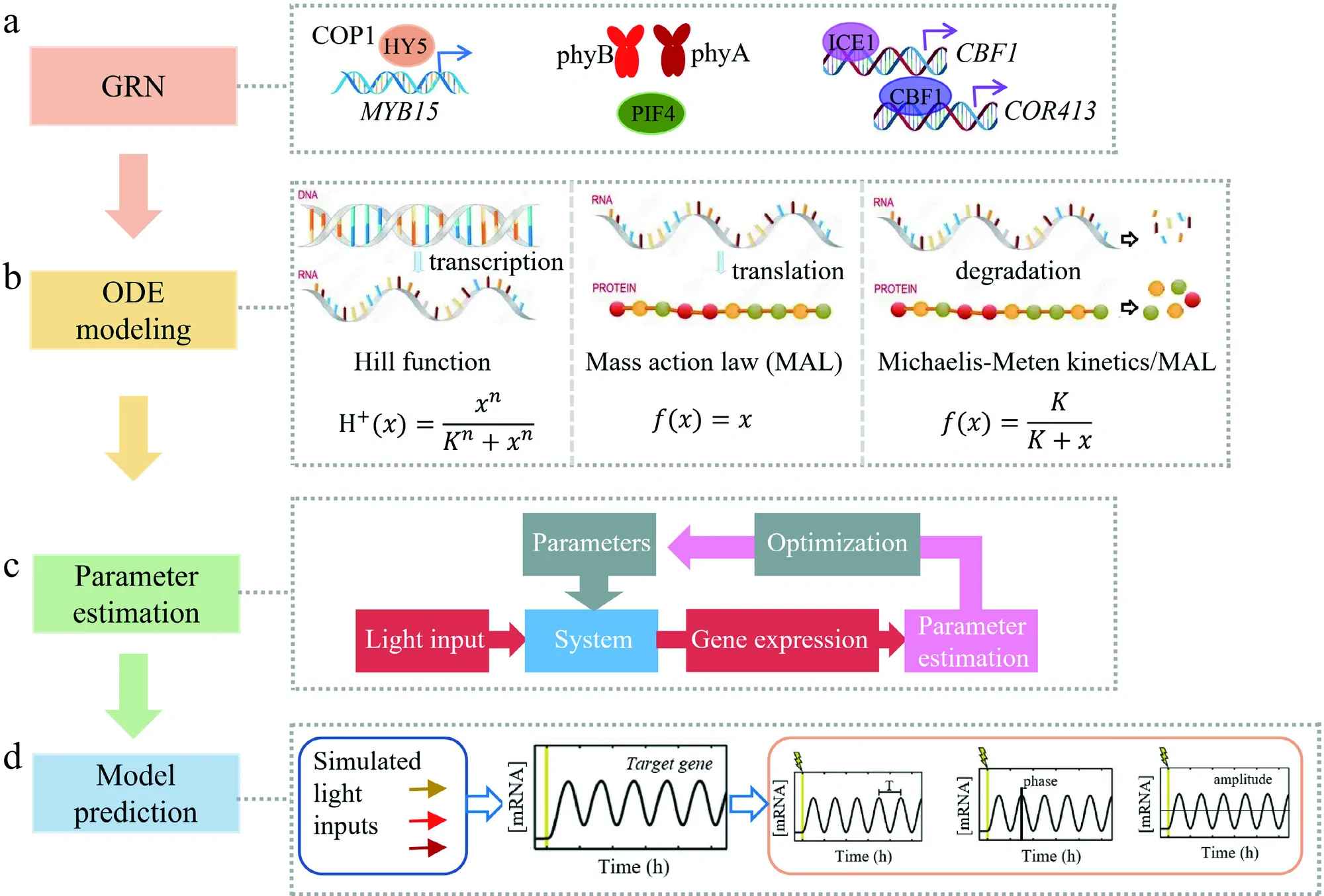
Modeling and verification process of the integrated regulatory network for light and low temperature signals. (a) Construction of the gene regulatory network (GRN). A core GRN was assembled from molecular evidence, integrating key light- and cold-responsive components. The network includes phyA/phyB photoreceptors (inhibiting PIF4 and HY5), COP1-regulated SlMYB15 activation, ICE1-mediated induction of CBF1, and downstream regulation of COR413, establishing a mechanistic foundation for mathematical formulation. (b) The ODE model's formulation. Biological processes were translated into quantitative kinetic equations: Transcriptional regulation was modeled using Hill functions to capture transcription factors' (TFs') dose–response patterns. Translation was described by mass-action kinetics; protein degradation was followed Michaelis–Menten or first-order decay. These components were integrated into an ODE system for a dynamic analysis of signal integration. (c) Parameter estimation. Model parameters were optimized by minimizing the sum of squared errors between simulated and experimental expression data obtained under 12-h light–12-h dark photocycles at 4 °C, including *SlHY5 *(wild-type) and *SlMYB15 *(wild-type, *hy5* mutant, and *SlHY5*-overexpression lines). Iterative least-squares fitting yielded a parameter set that accurately reproduces the observed dynamics. (d) Model prediction and experimental validation. Using the optimized parameters, the model was simulated under varied photoperiods. It successfully predicted the cycle length, phase, and amplitude changes of target genes, validating its capacity to simulate light–temperature signals' integration and providing a predictive tool for dissecting cold acclimation mechanisms under complex environmental scenarios.

The molecular basis of chilling response differs between tomato and *A. thaliana*, leading to species-specific regulation within the cold signaling pathway. First, althoguh MYB15 acts as a repressor of *CBF3* in the *Arabidopsis*-based H2025 model, it functions as an activator of *SlCBF1* in tomato. Experimental evidence supports this role: Zhang et al. demonstrated through electrophoretic mobility shift assays (EMSAs) that SlMYB15 directly binds to *SlCBF1* promoters *in vitro*^[11]^, a finding later confirmed *in vivo* by chromatin immunoprecipitation coupled with quantitative polymerase chain reaction (ChIP-qPCR). Dual-luciferase assays also showed that SlMYB15 significantly activates *SlCBF1*'s promoter activity under cold stress. Second, the regulation of *CBF* by ICE1 has been refined to reflect recent studies indicating that both phosphorylated and nonphosphorylated forms of ICE1 can bind to *CBF* promoters in both *A. thaliana* and tomato, as supported by genome-wide chromatin immunoprecipitation sequencing (ChIP-seq) data^[24,25]^. Third, to better integrate light and low-temperature signaling, the tomato model has been extended to include the COP1–HY5 regulatory axis and the PHY–PIF4–GIA4 module, capturing known photothermal crosstalk in cold acclimation. Figure 2 outlines the detailed procedure.

To assess quantitative differences in dynamic behavior between the two models, we ran both the tomato model and the H2025 *A. thaliana* model under identical environmental inputs (constant light [LL] at 4 °C). The temporal expression dynamics of key cold-responsive genes were simulated and compared with experimental measurements across the two plant stress models (Supplementary Fig. S2). In the *A. thaliana* H2025 model, the simulated *AtCBF3* transcript peaked at 1.87 h, compared with an experimental estimate of 3.19 h (Panel A). The simulated *AtCOR15A* levels gradually increased to a steady-state value of 2.63, mirroring the sustained upward trend observed experimentally (Panel B). For the tomato cold-acclimation model, the simulated *SlCBF1* expression reached its maximum at 1.97 h, and the overall time course was consistent with the fluctuating experimental measurements (Panel C). The simulated *SlCOR413* transcript abundance slowly approached a plateau at a steady-state value of 1.34 over the time course (Panel D). Overall, the model-derived kinetics, including peak timing and steady-state expression levels, showed better agreement with the experimental data for tomato than for *Al thaliana*, confirming that the constructed gene regulatory network models faithfully capture core cold-responsive transcriptional dynamics, with superior accuracy in the tomato system.

### Simulation and experimental validation of gene expression in response to cold shock

**Figure 3:**
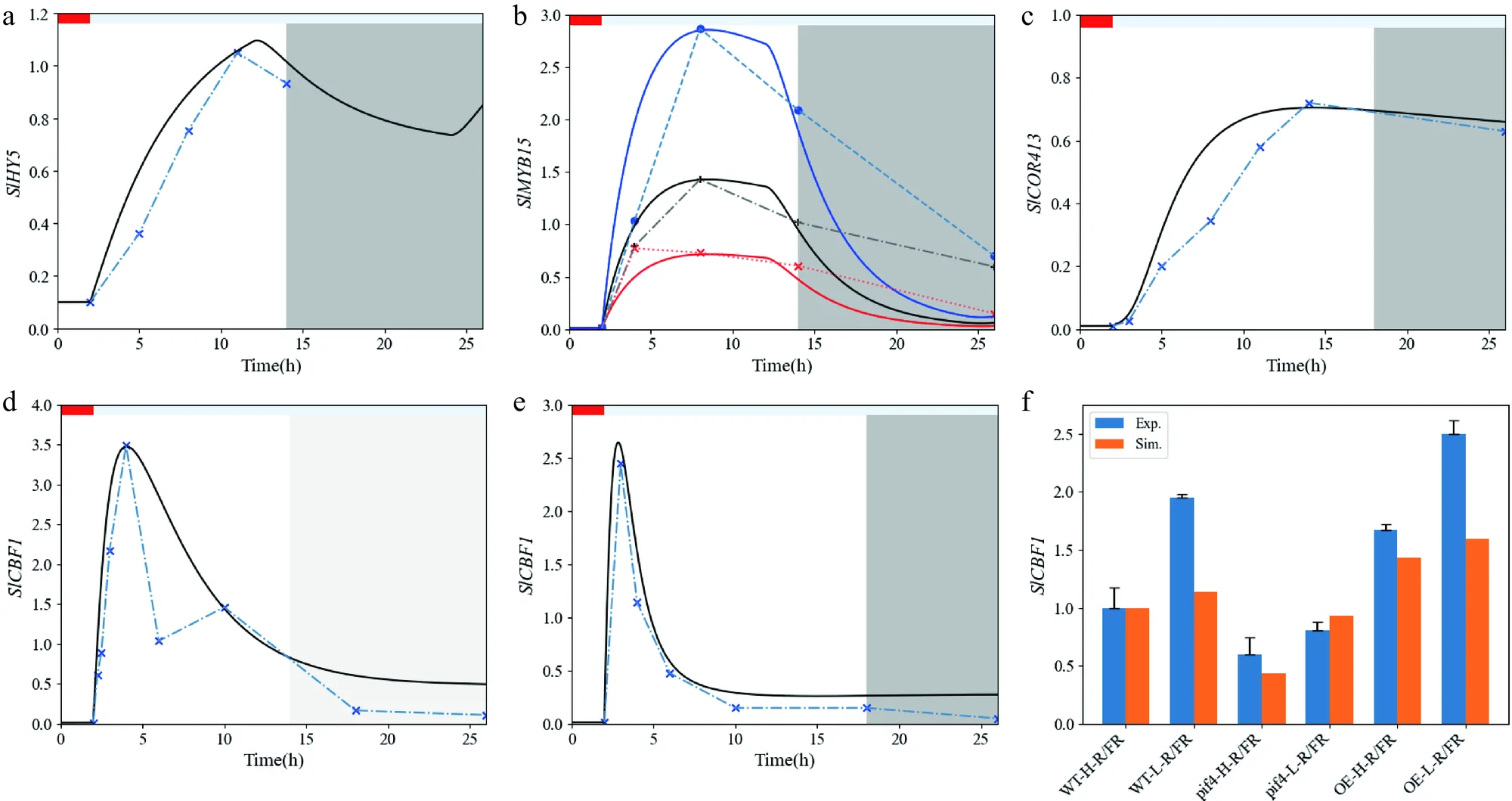
Time series validation of the cold-related genes after cold shock. (a, b) The expression levels of *SlHY5* and *SlMYB15* for basic parameter estimation. (c–e) Model validation of *SlCBF1* and *SlCOR413* expression in under light–dark (LD) or in LL conditions. (f) Experimental and simulated *SlCBF1* gene relative expression in tomato (wild-type [WT], *pif4* mutant, and SlPIF4-overexpressing [OE]) plants after exposure to 4 °C under H-R:FR or L-R:FR conditions. Experimental data (blue crosses) were derived from Figure 6a in Wang et al.^[14]^, from Figure 3b in Zhang et al.^[13]^, from Figure 2a in Zhang et al.^[12]^, and from Figure 2c in Wang et al.^[29]^. Black solid curves indicate the simulations, which were obtained by numerical integration of the kinetic equations (Eqs [1]–[24]), by the classical fourth-order Runge–Kutta method. The parameters are shown in Supplementary Table S2. The red and the blue bars represent warm and low temperature treatments, respectively.

On the basis of the expression data of *SlHY5* and *SlMYB15* across different genotypes^[13]^, we first estimated the model parameters by minimizing the squared error between the simulated and experimentally measured relative expression values, obtaining an optimized parameter set (Supplementary Table S2). The simulation captured the induction of *SlHY5*'s transcription after 3 h of cold exposure and its peak accumulation around 9 h under a 12-h photoperiod (Fig. 3a). Model-generated *SlMYB15* dynamics showed the highest expression in *SlHY5*-overexpressing plants and the lowest in *hy5* mutants, consistent with genotype-specific differences (Fig. 3b). These results confirm *SlHY5* as an upstream transcriptional regulator of *SlMYB15* during cold response. The model also accurately reproduced peak timing: *SlMYB15* peaked at ~6 h and *SlHY5* at 9–10 h after cold stress. As shown in Supplementary Table S3, the model exhibited high predictive accuracy for both training and validation datasets. The *R*^2^ values ranged from 0.836 to 0.918 for the training genes (*SlHY5* and *SlMYB15*) and from 0.843 to 0.986 for the validation genes (*SlCBF1* and *SlCOR413*), with correspondingly low RMSE values. These results confirm that the model simulations closely match the experimental observations across different genotypes and experimental conditions.

To accurately simulate the low-temperature regulation of *CBF* and *COR* genes, the model must recapitulate cold-induced expression dynamics. Using the established framework, we simulated the *SlCBF1* and *SlCOR413* profiles under cold shock under light–dark (LD) and LL conditions (Fig. 3c–e). Upon a drop in temperature to 4 °C (*t* = 2 h), *SlCBF1* rose rapidly under LD but more gradually under LL, whereas *SlCOR413* increased steadily under both photoperiods. Additionally, the model reproduced low red/far red (R:FR)-induced upregulation of *SlCBF1* and *SlCOR413* in wild-type and SlPIF4-overexpressing plants, but not in *pif4* mutants (Fig. 3f).

Simulations further showed that *SlCBF1* transcription peaked about 1 h after cold onset under LD (1 h earlier than under LL) and declined after ZT10 to a stable level above the baseline, consistent with experimental observations of rapid, light-period-modulated *SlCBF1* induction. The model also reproduced *SlCOR413*'s expression dynamics, namely immediate cold-induced upregulation, a peak at ~12 h, and a gradual decline within 24 h, matching reported data^[13]^. Together, these simulations confirm that the tomato cold-acclimation model reliably predicts the photoperiod-dependent timing of *SlCBF1*'s response and the sustained induction pattern of *SlCOR413*, validating its utility for dissecting light–temperature interplay in cold-responsive gene regulation.

To facilitate comparison of parameters between the two models, we screened 10 parameter pairs with large order-of-magnitude differences and conducted a parameter sensitivity analysis. As shown in the parameter sensitivity analysis (Supplementary Fig. S1), the model exhibited high sensitivity to certain parameters (particularly those related to transcriptional activation and protein synthesis), which could only maintain dynamic behavior within a narrow perturbation range to meet the criteria. In contrast, most other parameters had wider tolerance intervals, indicating that the model was generally insensitive to uncertainties in these parameters. This result clearly identifies the key regulatory nodes in the model that require precise experimental determination to ensure prediction accuracy.

### Temperature, photoperiod, and light quality shape phytochromes' dynamics to modulate low R:FR-induced cold tolerance

Although Wang et al. reported the antagonistic roles of phyA and phyB in regulating cold tolerance in tomato^[26]^, the dynamics of phytochrome activity and their direct interplay with CBF signaling at low temperatures remain unclear. In *A. thaliana*, recent studies have indicated that phyB enhances freezing tolerance through direct interaction with CBFs within a CBF–phyB–PIF regulatory module, where CBFs bind *PIF3* to inhibit the co-degradation of PIF3 and phyB, thereby stabilizing phyB to promote stress-responsive gene expression^[27,28]^.

**Figure 4:**
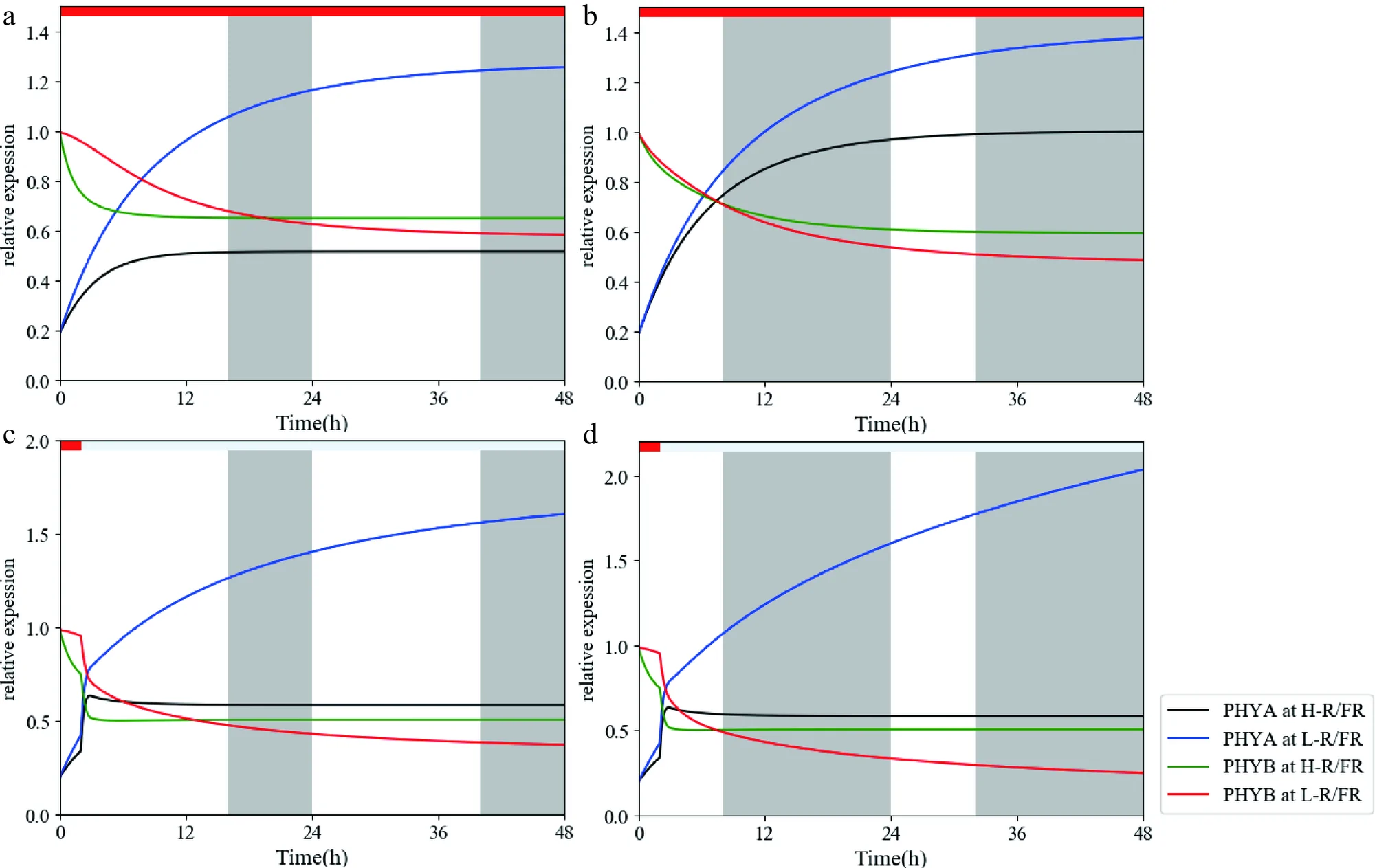
Tomato phytochromes in response to variations in temperature, photoperiod, and light quality. (a–d) Accumulation of phytochrome proteins (SlPHYA; SlPHYB) as influenced by temperature, photoperiod and light quality in tomato plants. Plants were maintained at 25 or 10 °C under LD (16 h) or SD (8 h) conditions with a high R:FR ratio (* γ =0.5 *) or a low R:FR ratio (* γ =2.5 *). Numerical simulation was carried out with the light input function * L(t) *, the cold signal input variable CaM, and the R:FR ratio * γ * in our mathematical model.

In tomato, combined short-day (SD; 8 h) and low R:FR conditions optimally induce transcription of *SlPHYA* while suppressing *SlPHYB*, whereas light quality and photoperiod exert a minimal influence on *SlCBF1*'s expression at 25 °C compared with cold acclimation^[29]^. To test these observations computationally, we simulated the phytochrome dynamics under varying R:FR ratios (*γ* = 0.5 for high R:FR, *γ* = 2.5 for low R:FR) and photoperiods defined by the light function *L(t)*. Temperature transitions were modeled using a modified Plieth equation, decreasing gradually from 25 to 10 °C. Simulations confirmed that both at 25 and 10 °C, SD and low R:FR promote the accumulation of SlPHYA and reduce SlPHYB levels, with cold exposure further amplifying this trend, especially under SD and low R:FR conditions (Fig. 4).

To assess phytochrome proteins' stability under cold stress, we simulated phyA and phyB levels using one- and two-compartment Ca^2^^+^ models (Supplementary Fig. S3), which are widely known^[15,30,31]^. Although both models predicted a slight post-cold decline in phyB before stabilization, phyA levels rose transiently and then settled at a steady state higher than that of phyB. These results align with experimental reports in *A. thaliana*, suggesting that cold treatment enhances phyA/phyB proteins' stability, thereby supporting improved cold tolerance.

**Figure 5:**
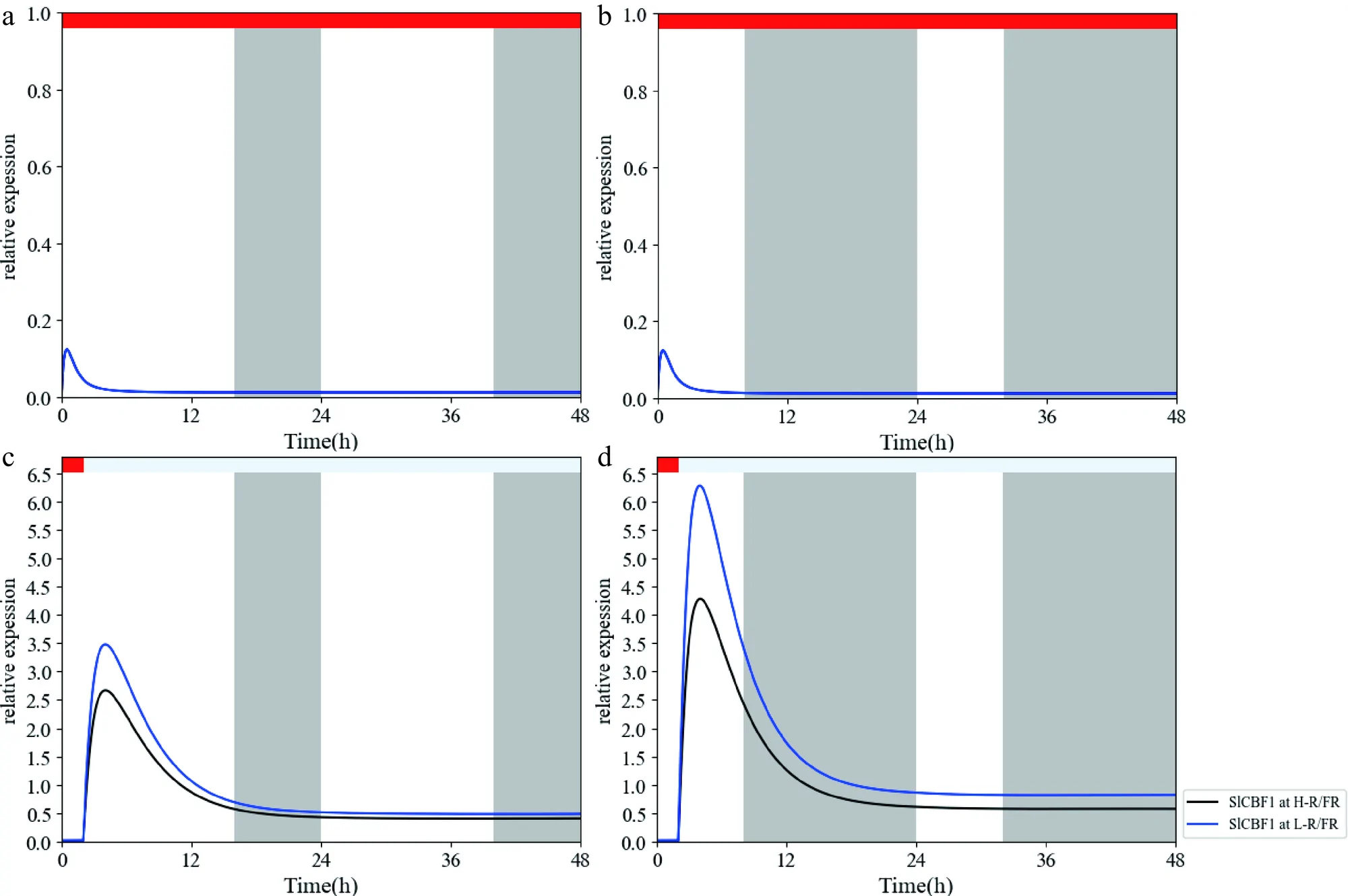
Short-day (SD) and low R/FR-induced cold tolerance in tomato plants. (a–d) Transcripts of the cold-tolerant gene *SlCBF1* as influenced by temperature, photoperiod, and light quality in tomato plants. Plants were maintained at 25 or 10 °C under LD (16 h) or SD (8 h) conditions with a high R:FR ratio (* γ =0.5 *) or a low R:FR ratio (* γ =2.5 *). Numerical simulation was carried out with the light input function * L(t) *, the cold signal input variable CaM, and R:FR ratio * γ * in our mathematical model. Whereas light quality and photoperiod had little effect on the transcription of *SlCBF1* in plants grown at 25 °C (see parts a and b), low temperature (10 °C) induced the transcription of *SlCBF1*, especially under SD and low R:FR conditions.

Figure 5 further corroborates that SD and low R:FR collectively enhance cold tolerance in tomato. At 25 °C, neither photoperiod nor light quality substantially affected *SlCBF1*'s transcription. In contrast, low temperature combined with SD and low R:FR strongly induced the expression of *SlCBF1*, with sustained cold leading to significantly higher steady-state *SlCBF1* levels under SD/low R:FR than under long-day/high R:FR conditions.

### Oscillation of SlPIF4 is induced by the negative feedback loop of PIF4–GAI4

**Figure 6:**
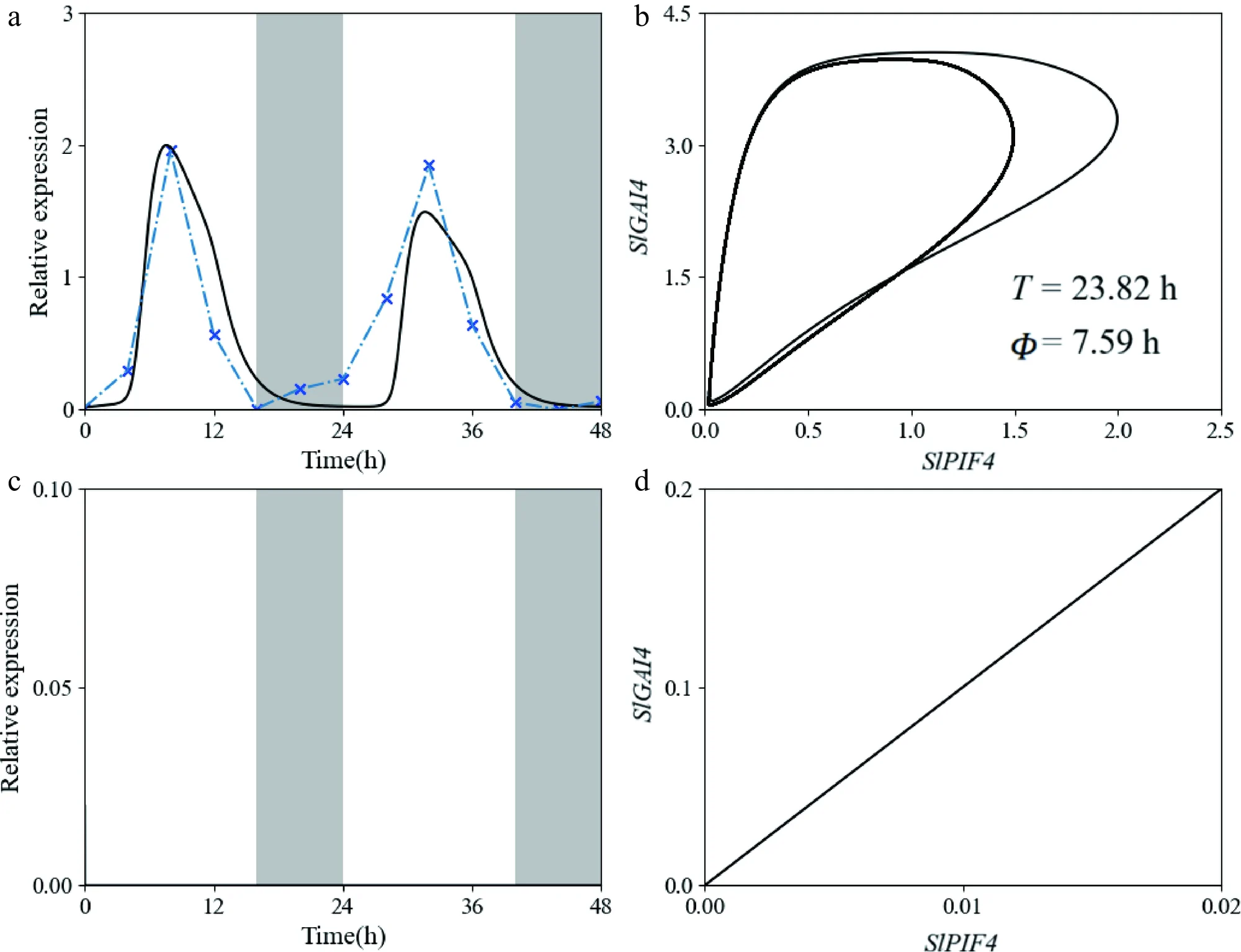
The PIF4–GAI4 negative feedback loop regulates the oscillation of *SlPIF4's* expression. (a) Gray and white backgrounds denote dark (12 h) and light (12 h) periods, respectively. Blue dotted line with asterisks: Experimental relative expression of PIF4; black solid line: model simulation. (b) The closed orbit reflects a stable limit cycle generated by negative feedback between *PIF4 *mRNA and GAI4 protein, with a period of 23.82 h and a phase lag of 7.59 h. (c) When the negative feedback interaction is removed, the expression of *PIF4* loses rhythmicity and remains near the baseline, failing to respond to light–dark transitions. (d) The system shifts from a closed orbit to a monotonically increasing linear path, indicating a transition from oscillatory to steady-state dynamics.

Through integrated modeling and experimental validation, we identified the PIF4–GAI4 negative feedback loop as the core mechanism driving the rhythmic expression of *PIF4*. Under a 12-h light:12-h dark photocycle, both experimental measurements and model simulations displayed closely matched diurnal oscillations of *SlPIF4*'s transcript levels (Fig. 6a). After dawn, the expression of *SlPIF4* rose rapidly and peaked near midday, then declined sharply after dusk to near-baseline levels, forming a stable periodic pattern. The simulated trajectory (black solid line) closely aligned with the experimental profile (blue dotted line and asterisks), confirming the model's accuracy in capturing this dynamic.

Phase-plane analysis of the dynamic relationship between *SlPIF4 *and SlGAI4 protein revealed a closed periodic orbit (Fig. 6b) with a period of 23.82 h and a phase delay of 7.59 h. This closed trajectory reflects the underlying regulatory logic: SlPIF4 activates the transcription and translation of *SlGAI4*, but increased SlGAI4 protein subsequently represses *SlPIF4*'s expression. Degradation of GAI4 then releases this repression, allowing transcription of *SlPIF4* to restart, thereby sustaining stable oscillations^[32]^.

To test the functional necessity of this feedback loop, we removed the PIF4–GAI4 interaction from the model. In the absence of feedback, *SlPIF4*'s expression lost all rhythmicity, remaining near zero throughout the light–dark cycle (Fig. 6c). The corresponding phase plot shifted from a closed orbit to a monotonically increasing linear path (Fig. 6d), indicating a transition from oscillatory to steady-state dynamics. These results directly demonstrate that the PIF4–GAI4 negative feedback circuit is essential for generating and maintaining rhythmic expression of *SlPIF4*; without it, the system fails to oscillate and exhibits only a simple, attenuated response.

### Oscillation of *SlCOR413* is induced by low temperature

*COR* genes are known to respond to cold stress, and their products play important roles in cold acclimation^[33]^. Enhanced chilling tolerance in transgenic lines is often associated with the induction of *CBF* and *COR* genes^[33,34]^. Fluctuating temperatures influence freezing tolerance, primarily through desensitization and resensitization mechanisms that involve the dynamic regulation of CBF's and COR's expression^[35]^.

**Figure 7:**
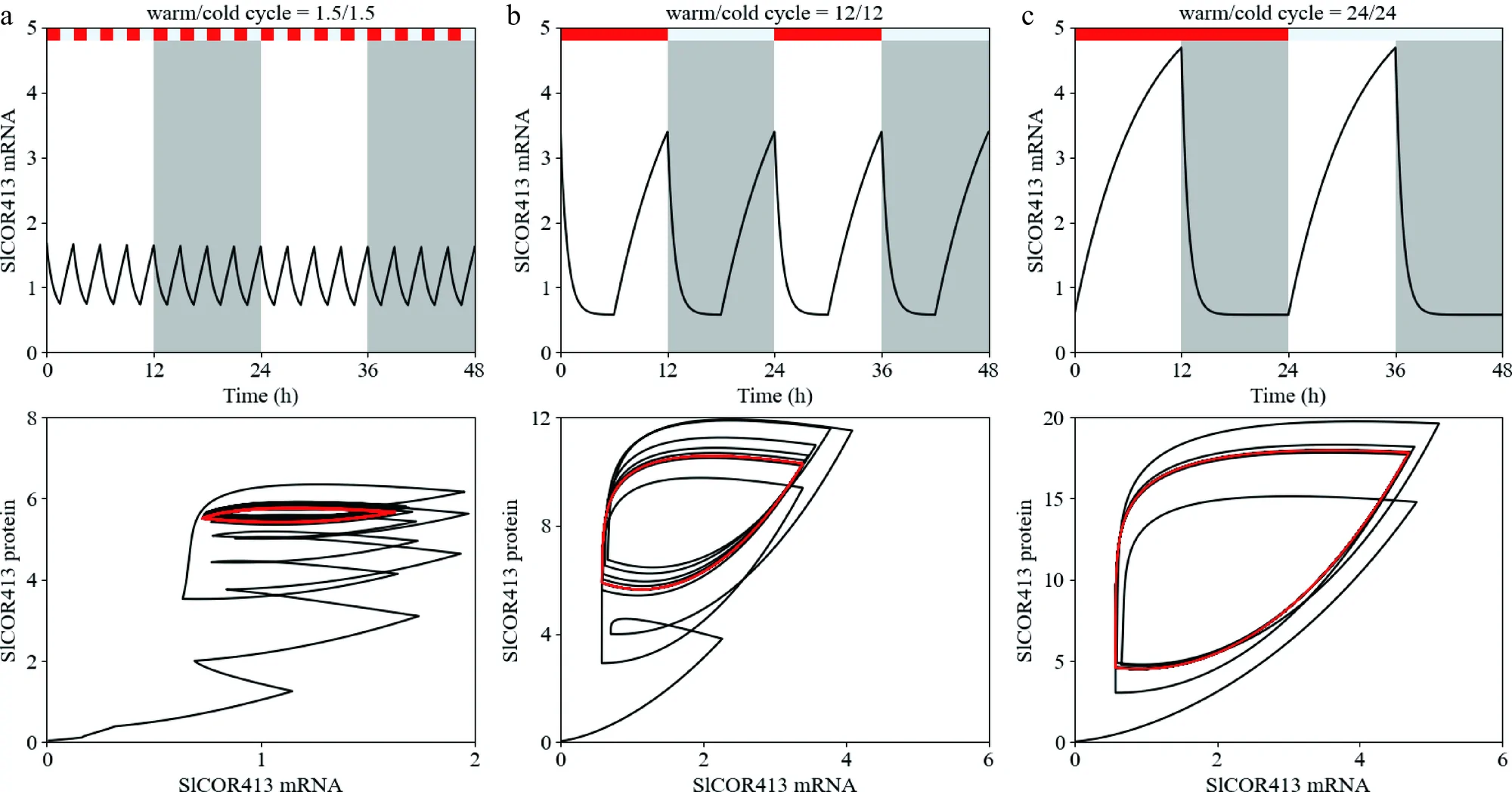
Cold-induced oscillation of *SlCOR413* in tomato plants. In order to investigate the oscillation of cold-related genes induced by low temperature, the dynamic behavior of plants subjectively simulated under continuous low temperature after five rounds of different cooling and warming cycles (warm:cold = 1.5:1.5 in Panel a; warm:cold = 12:12 in Panel b; warm:cold = 24:24 in Panel c).

Beyond direct induction of cold-related genes, coupling between the circadian clock and cold response pathways may further support plants' growth and survival under low temperatures^[36]^. This raises the question of whether cold-related genes can generate self-sustained oscillations that enhance acclimation even in the absence of circadian input. To investigate this, we exposed tomato plants to repeated warm–cold cycles followed by continuous low temperature (4 °C) under a 12-h light/12-h dark photoperiod. We focused on *SlCOR413*'s dynamics, modeled as driven by a sequence of Ca^2^^+^ pulses, and quantified its mRNA profiles under different warm–cold regimes: 1.5 h/1.5 h (Fig. 7a), 12 h/2 h (Fig. 7b), and 24 h/24 h (Fig. 7c).

Simulations showed that upon cessation of the temperature cycles, *SlCOR413*'s transcript levels dropped to a steady state and then exhibited sustained, robust oscillations. The period and amplitude of these oscillations depended strongly on the duration of the preceding warm–cold cycles, with longer cycles resulting in higher amplitudes. Notably, under a 24 h/24 h regime, *SlCOR413* mRNA displayed an intrinsic circadian-like rhythm with a period close to 24 h, indicating that temperature cycles alone can entrain a persistent oscillatory response in this cold-responsive gene.

The peak expression time of *SlCOR413* exhibited a logarithmic increase with extension of the temperature cycle's duration (Supplementary Fig. S4). When the temperature cycle increased from 2 to 12 h, the peak time rapidly delayed from approximately 3 to 12 h; as the cycle extended further to 24 h, the rate of delay slowed down. The logarithmic fitting equation was * y=4.6269łn (x)-1.5618 *, with a high coefficient of determination (*R*^2^ = 0.8988), confirming a significant logarithmic correlation between the temperature cycle's duration and peak time. This result indicates that the time of *SlCOR413*'s peak expression does not synchronize linearly with the temperature cycle, but instead shows a logarithmically dependent delayed response, reflecting a nonlinear adaptation mechanism of gene expression to environmental cycles.

Meanwhile, the rhythmic period and amplitude of *SlCOR413* showed an extremely strong linear positive correlation with the temperature cycle's duration (Supplementary Fig. S5), with the fitting equations* y=0.9349x+1.3136 * (*R*^2^ = 0.9981) and * y=0.0771x+1.0462 * (*R*^2^= 0.9939), respectively. This demonstrates that the rhythmic period of *SlCOR413* almost perfectly synchronizes with the temperature cycle, highlighting the plant's precise ability to track environmental cycles. However, the slope of the increase in amplitude (0.0771) was significantly smaller than that of increase in period (0.9349), indicating that the regulatory effect of the temperature cycle's duration on the rhythmic period is far stronger than that on amplitude. This difference reflects a robust rhythmic adaptation strategy: When responding to changes in the environmental cycle, plants prioritize maintaining the stability of the rhythmic period, but changes in amplitude remain relatively moderate.

## Discussion

Plants perceive low temperatures and respond by integrating photoperiod, light quality, and thermal stimuli to ensure the correct induction of cold tolerance genes^[37]^. For nearly two decades, research has predominantly focused on dissecting the components and molecular mechanisms of the cold signaling pathway, particularly the CBF-dependent cascade^[6,38,39]^. However, the regulatory roles of phytochromes or cryptochromes in cold acclimation have received comparatively less attention. Recent work shows that *PHYB* is essential for the ability of trees to maintain growth under lower temperatures in permissive long days^[40]^. Most research was focused on *A. thaliana*, such as the reduced transcript levels of *CBF* and *COR* genes after exposure to blue light^[41]^. The TF *SlWRKY2* was identified as a critical hub linking phytochrome-mediated light perception with cold stress adaptation in tomato^[42]^. Drawing on recent experimental data in tomato, we have refined the architecture of the cold and light signaling pathway and developed a mathematical model that demonstrates strong correspondence with a broad spectrum of both newly generated and previously published data. The model systematically recapitulates tomato's responses to diverse environmental perturbations and offers an integrated framework for understanding the cold-responsive gene regulatory network (Fig. 1).

The most significant advancement of our model relative to the the H2025 framework of *A. thaliana* lies in the explicit incorporation of light signals, both photoperiod and light quality, into the cold response system (Fig. 2). This integration is biologically meaningful because cold stress in natural environments rarely occurs in isolation; rather, it is accompanied by seasonal changes in daylength and light's spectral composition. Our simulations demonstrate that photoperiod profoundly modulates cold-induced gene expression, consistent with observations that cold tolerance varies across photoperiodic treatments^[8]^. Light signals, perceived through the phytochrome, cryptochrome, and phototropin photoreceptor families, are indispensable for cold acclimation^[29]^. By mimicking variations in daylength and photoreceptor activity, our model captures how plants coordinate multiple environmental cues to optimize cold tolerance—a feature that single-factor models cannot address.

The COP1–HY5–MYB15 module, incorporated as an upstream hub in our cold tolerance pathway, represents a mechanistically grounded innovation. Evidence indicates that the transcription levels of *SlHY5* and *SlCOP1* are strongly influenced by yjr photoperiod and light quality under low-temperature conditions^[29]^. HY5 has been shown to positively regulate the expression of *MYB15* and *CBF* while co-regulating CBF-dependent cold tolerance with MYB15^[11]^. Our simulations confirm *SlHY5* as an upstream transcriptional regulator of *SlMYB15* during the cold response, and the model successfully reproduces the genotype-specific expression patterns observed in SlHY5-overexpressing and *hy5* mutant plants. The functional divergence of MYB15 homologs across species was noted. AtMYB15 negatively regulates cold tolerance in *A. thaliana*^[43]^, whereas SlMYB15 acts as a positive regulator in tomato^[11]^, underscoring the importance of species-specific modeling and cautions against direct extrapolation of findings from *A. thaliana *to crop species. The COP1–HY5–MYB15 module, by accurately reflecting this positive regulatory role of SlMYB15, enriches our understanding of the molecular mechanisms underlying cold response in tomato and highlights the evolutionary plasticity of cold signaling networks.

To our knowledge, this study represents the first mathematical modeling effort to systematically examine how specific phytochromes regulate cold stress responses in tomato. By simulating time-series dynamics of photoreceptor proteins, we were able to monitor their behavior under varying environmental conditions. Our model successfully reproduces the experimentally observed increase in transcription of *SlPHYA* and the concomitant decrease in the transcription of *SlPHYB* in response to declining temperatures, shortened daylength, and reduced R:FR ratios, followed by elevated *SlCBF1* expression and enhanced cold tolerance (Figs 3 and 4). These simulations suggest that mathematical modeling provides a robust approach for tracking phytochrome-dependent adaptive mechanisms that enable plants to cope with fluctuations in temperature, photoperiod, and light quality. The ability to predict phytochrome dynamics under multiple stress conditions represents a valuable tool for designing targeted experiments to validate specific regulatory nodes.

The PHY–PIF4 module, based on a proposed tomato cold-tolerance model integrating light and temperature signals^[32]^, is a central structure of our network. In this module, low R:FR ratios and low temperatures act as positive factors inducing *SlPIF4*'s expression, which, in turn, activates *SlCBF1* and *SlGIA4*. However, the functional roles of PIF homologs vary considerably across plant species. In *A. thaliana*, PIF4 and PIF7 negatively regulate *CBF* transcription to prevent the unnecessary activation of cold acclimation pathways^[44]^. PIF3 directly represses the expression of *CBF* by binding to *CBF* promoters in a phyB-dependent manner (Jiang et al., 2017.10). PIF1, PIF4, and PIF5, which are destabilized under cold stress, negatively regulate freezing tolerance^[28]^. These interspecific differences caution against generalizing regulatory mechanisms across taxa and underscore the value of our tomato-specific model.

To better understand the regulatory logic governing the expression of *SlCBF1*, we summarized its known TFs, including its activators (SlICE1, SlHY5, SlMYB15, SlCAMTA, SlPIF4) and repressors (SlZAT). Though most of these factors have been discussed above, CAMTA and ZAT merit additional consideration. CAMTA proteins have been studied primarily for their roles in defense-related responses against pathogens and drought tolerance^[45]^, but their involvement in cold stress responses remains incompletely understood. In *A. thaliana*, CAMTA proteins provide a positive link between Ca^2^^+^ signatures and cold acclimation^[46]^. We therefore incorporated the positive regulation of *SlCBF1* by CAMTA in light of the precedent of *A. thaliana*, though direct experimental validation in tomato would strengthen this assumption. Regarding ZAT proteins, ZAT12 represses *CBFs*, whereas ZAT10 activates *CORs*^[47]^. Because zinc finger proteins are not core components of our network's architecture, we simplified the model by integrating ZAT12 and ZAT10 into a single variable. This simplification, though reducing the model's complexity, may obscure distinct regulatory roles and should be revisited if more detailed data become available.

The ICE1–CBF–COR module remains the central axis of the cold signaling network, with its components interacting cooperatively to induce cold resistance. The transcription levels of *CBFs* and *CORs* serve as quantitative markers of cold-responsive capacity. A series of activators and repressors positively or negatively regulate these genes, forming a complex cold signaling pathway. Cold tolerance in plants is a complex quantitative trait, typically occurring in response to combinations or successions of environmental stresses (Fig. 5). Therefore, the combined influences of multiple factors must be considered within a dynamic systems framework. Importantly, cold acclimation is not governed solely by the CBF-dependent pathway; CBF-independent pathways, including circadian clock regulation and hormone signaling, also exert significant influence^[48]^. The relative contributions of these parallel pathways to overall cold tolerance, and how they interact with the CBF core, remain open questions that our model can help address through further perturbation analyses.

A particularly intriguing finding from our modeling is the predicted oscillatory behavior of the expression of *SlPIF4* (Fig. 6). Experimental data reveal a clear diurnal expression profile of *SlPIF4* in leaves, with transcript levels exhibiting periodic oscillation. However, our baseline network, which incorporates light input and light quality signals but lacks an explicit circadian clock component, cannot generate sustained oscillations. To overcome this limitation, we appended a periodic sine function as a clock input signal to approximate the oscillatory dynamics. This approach, though functionally effective, represents a phenomenological rather than mechanistic treatment of circadian regulation. The circadian clock components ELF3 and TOC1 are known to regulate *PIF4*'s expression. ELF3 represses mRNA levels of *PIF4*, and warm temperatures relieve this repression by inhibiting ELF3's binding to the *PIF4* promoter, whereas TOC1 interacts with and suppresses *PIF4*^[49]^. Incorporating these clock components explicitly into future model iterations would provide a more mechanistically grounded representation of *SlPIF4*'s oscillation and its coupling to cold signaling.

There are several intrinsic limitations to our modeling framework. First, the 80 parameters in our model were estimated from a relatively limited set of experimental data, which introduces uncertainty into certain predictions. Parameters' identifiability may be compromised by data sparsity, and some parameter values may not be uniquely determined. Future studies using more extensive time-series data under standardized conditions would improve parameter estimation and reduce the predictions' uncertainty. Second, the predicted oscillatory behavior of *SlCOR413*'s transcript abundance (Fig. 7) has not yet been experimentally verified. Although our model generates this oscillation on the basis of the integrated regulatory structure, direct experimental confirmation through high-resolution time-series measurements under controlled conditions would substantially strengthen the robustness of this prediction.

Additionally, the comparability of datasets obtained under different experimental conditions deserves careful consideration. All tomato expression data used in this study were derived from experiments conducted under controlled environmental conditions with documented light and temperature regimes, which ensures a reasonable degree of reproducibility. Nevertheless, cross-dataset comparisons should be interpreted with caution, especially when experimental details such as photoperiod, cold treatment intensity, or tissue sampling time are not fully harmonized. The model is primarily designed to capture relative trends in cold-responsive genes' expression rather than to predict absolute expression values, so baseline differences between experiments have a relatively limited impact on the model's validation. However, future efforts to standardize experimental protocols across laboratories would greatly facilitate more rigorous model testing and cross-study comparisons.

## Conclusion

In this study, our integrated computational model successfully recapitulates the dynamic crosstalk between light and cold signaling pathways in tomato, with experimental validation across distinct photoperiod regimes. Beyond reproducing documented gene expression patterns, the model generates testable predictions: Elevated *CBF* expression under low red/far-red light, the enhanced stability of phyA and phyB at low temperatures, and the oscillatory dynamics of *SlPIF4* and *SlCOR413* upon cold exposure. These findings identify phytochrome activity and the SlPIF4–SlGAI4 negative feedback loop as core regulatory hubs governing cold tolerance. This modeling framework serves as a predictive tool to dissect light–cold crosstalk, laying a rational foundation for engineering cold-tolerant horticultural varieties via targeted transcriptional manipulation. Future experimental validation of these predicted oscillatory kinetics will further improve the model's performance and expand its translational utility.

## SUPPLEMENTARY DATA

Supplementary data to this article can be found online.

## Data Availability

This study did not generate new experimental data. All data used in the analyses were obtained from previously published studies, which are cited in the manuscript. The original datasets are available from the corresponding authors of those studies upon reasonable request.

## References

[1] (2022). Calcium mediated cold acclimation in plants: underlying signaling and molecular mechanisms. Frontiers in Plant Science.

[2] (2026). Advances in the study of the role of epigenetic mechanisms 3 in plant growth and response to stress. Horticulture Research.

[3] (2026). Decoding the cold signal: hormonal networks orchestrating the trade-off between growth and tolerance. Tropical Plants.

[4] (2026). Circadian clock regulation of low R/FR light signaling and photosynthetic cross-talk in crop development. Environmental and Experimental Botany.

[5] (2026). Integrated circadian regulation in horticultural plants: light-environment mechanisms governing growth and development. Horticulture Research.

[6] (2018). Molecular regulation of CBF signaling in cold acclimation. Trends in Plant Science.

[7] (2020). Advances in AP2/ERF super-family transcription factors in plant. Critical Reviews in Biotechnology.

[8] (2012). Photoperiodic regulation of the C-repeat binding factor (CBF) cold acclimation pathway and freezing tolerance in *Arabidopsis thaliana*. Proceedings of the National Academy of Sciences of the United States of America.

[9] (2020). Molecular regulation of plant responses to environmental temperatures. Molecular Plant.

[10] (2020). COLD-REGULATED GENE27 integrates signals from light and the circadian clock to promote hypocotyl growth in *Arabidopsis*. The Plant Cell.

[11] (2020). The HY5 and MYB15 transcription factors positively regulate cold tolerance in tomato via the CBF pathway. Plant, Cell & Environment.

[12] (2004). Freezing-sensitive tomato has a functional CBF cold response pathway, but a CBF regulon that differs from that of freezing-tolerant *Arabidopsis*. The Plant Journal.

[13] (2021). Cold-regulated gene *LeCOR413PM2* confers cold stress tolerance in tomato plants. Gene.

[14] (2017). *SlMAPK1/2/3* and antioxidant enzymes are associated with H_2_O_2_-induced chilling tolerance in tomato plants. Journal of Agricultural and Food Chemistry.

[15] (1999). Temperature sensing by plants: calcium-permeable channels as primary sensors − a model. The Journal of Membrane Biology.

[16] (1999). Temperature sensing by plants: the primary characteristics of signal perception and calcium response. The Plant Journal.

[17] (2010). Breaking the code: Ca^2+^ sensors in plant signalling. Biochemical Journal.

[18] (2013). The tomato calcium sensor Cbl10 and its interacting protein kinase Cipk6 define a signaling pathway in plant immunity. The Plant Cell.

[19] (2018). Microspore embryogenesis in *Brassica*: calcium signaling, epigenetic modification, and programmed cell death. Planta.

[20] (2019). Calcium and calcium sensors in fruit development and ripening. Scientia Horticulturae.

[21] (2024). Calmodulins and calmodulin-like proteins-mediated plant organellar calcium signaling networks under abiotic stress. The Crop Journal.

[22] (2026). Calcium signaling in plants: universal and unique paradigms. Cell.

[23] (2025). A computational model for cold entrainment of the ICE1-CBF-COR pathway in *Arabidopsis*. Computational and Structural Biotechnology Reports.

[24] (2017). Different cold-signaling pathways function in the responses to rapid and gradual decreases in temperature. The Plant Cell.

[25] (2020). The transcription factor ICE1 functions in cold stress response by binding to the promoters of *CBF* and *COR* genes. Journal of Integrative Plant Biology.

[26] (2016). Phytochrome A and B function antagonistically to regulate cold tolerance via abscisic acid-dependent jasmonate signaling. Plant Physiology.

[27] (2017). PIF3 is a negative regulator of the *CBF* pathway and freezing tolerance in *Arabidopsis*. Proceedings of the National Academy of Sciences of the United States of America.

[28] (2020). Cold-induced CBF–PIF3 interaction enhances freezing tolerance by stabilizing the phyB thermosensor in *Arabidopsis*. Molecular Plant.

[29] (2019). SlHY5 integrates temperature, light, and hormone signaling to balance plant growth and cold tolerance. Plant Physiology.

[30] (1990). Minimal model for signal-induced Ca^2+^ oscillations and for their frequency encoding through protein phosphorylation. Proceedings of the National Academy of Sciences of the United States of America.

[31] (1991). Signal-induced Ca^2+^ oscillations: properties of a model based on Ca^2+^-induced Ca^2+^ release. Cell Calcium.

[32] (2020). Crosstalk of PIF4 and DELLA modulates CBF transcript and hormone homeostasis in cold response in tomato. Plant Biotechnology Journal.

[33] (2019). Cold acclimation by the CBF–COR pathway in a changing climate: lessons from *Arabidopsis thaliana*. Plant Cell Reports.

[34] (2015). Regulation of the Arabidopsis CBF regulon by a complex low-temperature regulatory network. The Plant Journal.

[35] (2003). Cold induction of Arabidopsis *CBF* genes involves multiple ICE (inducer of *CBF* expression) promoter elements and a cold-regulatory circuit that is desensitized by low temperature. Plant Physiology.

[36] (2015). Circadian regulation of abiotic stress tolerance in plants. Frontiers in Plant Science.

[37] (2026). Modeling the circadian period and phase shifts in tomatoes: low degradation-driven oscillator dynamics under continuous light and temperature. Journal of Theoretical Biology.

[38] (2021). The direct targets of CBFs: in cold stress response and beyond. Journal of Integrative Plant Biology.

[39] (2025). Regulation of alternative splicing by CBF-mediated protein condensation in plant response to cold stress. Nature Plants.

[40] (2025). Phytochrome B and phytochrome-interacting-factor4 modulate tree seasonal growth in cold environments. Nature Communications.

[41] (2021). Effects of the blue light–cryptochrome system on the early process of cold acclimation of *Arabidopsis thaliana*. Environmental and Experimental Botany.

[42] (2026). SlWRKY2 orchestrates cold tolerance via phytochromes interaction and SlPIF4 stabilization in tomato. The Plant Cell.

[43] (2006). A R2R3 type MYB transcription factor is involved in the cold regulation of *CBF* genes and in acquired freezing tolerance. Journal of Biological Chemistry.

[44] (2022). PIFs- and COP1-HY5-mediated temperature signaling in higher plants. Stress Biology.

[45] (2021). Calmodulin binding transcription activators: an interplay between calcium signalling and plant stress tolerance. Journal of Plant Physiology.

[46] (2020). Ca^2+^/calmodulin complex triggers CAMTA transcriptional machinery under stress in plants: signaling cascade and molecular regulation. Frontiers in Plant Science.

[47] (2024). A C2H2-type zinc finger protein ZAT12 of *Poncirus trifoliata* acts downstream of CBF1 to regulate cold tolerance. The Plant Journal.

[48] (2020). CBF-phyB-PIF module links light and low temperature signaling. Trends in Plant Science.

[49] (2016). Abiotic stress signaling and responses in plants. Cell.

