# Supplementary material for: Integrative modeling of cold and light signaling in tomato: unraveling the transcriptional network governing cold acclimation
Source: Eng Life Sci. 2026 Aug 12;26(8):e007. doi: 10.48130/els-0026-0007 (PMC13599643; doi:10.48130/els-0026-0007)
Supplement: Supplementary file 1 [file els-2026-26-8-0007-S1.zip › 10.48130_els-0026-0007-Suppl-FigureS1.pdf]

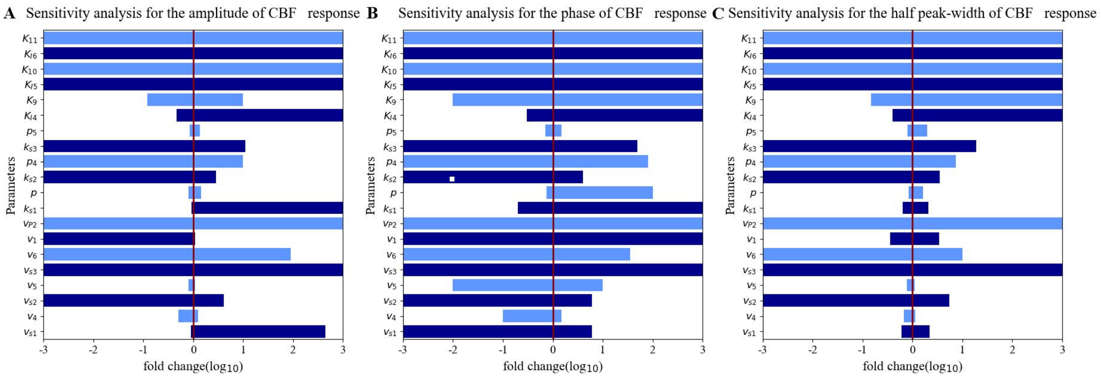

Supplementary Figure 1. Sensitivity analysis for the amplitude, phase and half peak-width of *CBF* mRNA exposed to cold stress under different plant stress models.

By comparing the parameters of the tomato model and the H2025 *Arabidopsis* model (Table S2), we selected 10 parameter pairs that exhibit obvious differences in magnitude and performed one-at-a-time parameter analysis on them. Each parameter was varied from 10^{-3} to 10^{3} times its baseline value (log scale, x-axis) while others were held invariable. Colored intervals indicate parameter sets that yield cold-induced *CBF* mRNA dynamics meeting three criteria: (A) peak amplitude around steady-state, (B) peak time 1–6 h, and (C) half-peak width (duration above 50% of peak) 3–6 h. Dark blue bars and light blue bars represent parameters in the *Arabidopsis* and tomato models, respectively.
