# Supplementary material for: Integrative modeling of cold and light signaling in tomato: unraveling the transcriptional network governing cold acclimation
Source: Eng Life Sci. 2026 Aug 12;26(8):e007. doi: 10.48130/els-0026-0007 (PMC13599643; doi:10.48130/els-0026-0007)
Supplement: Supplementary file 1 [file els-2026-26-8-0007-S1.zip › 10.48130_els-0026-0007-Suppl-FigureS2.pdf]

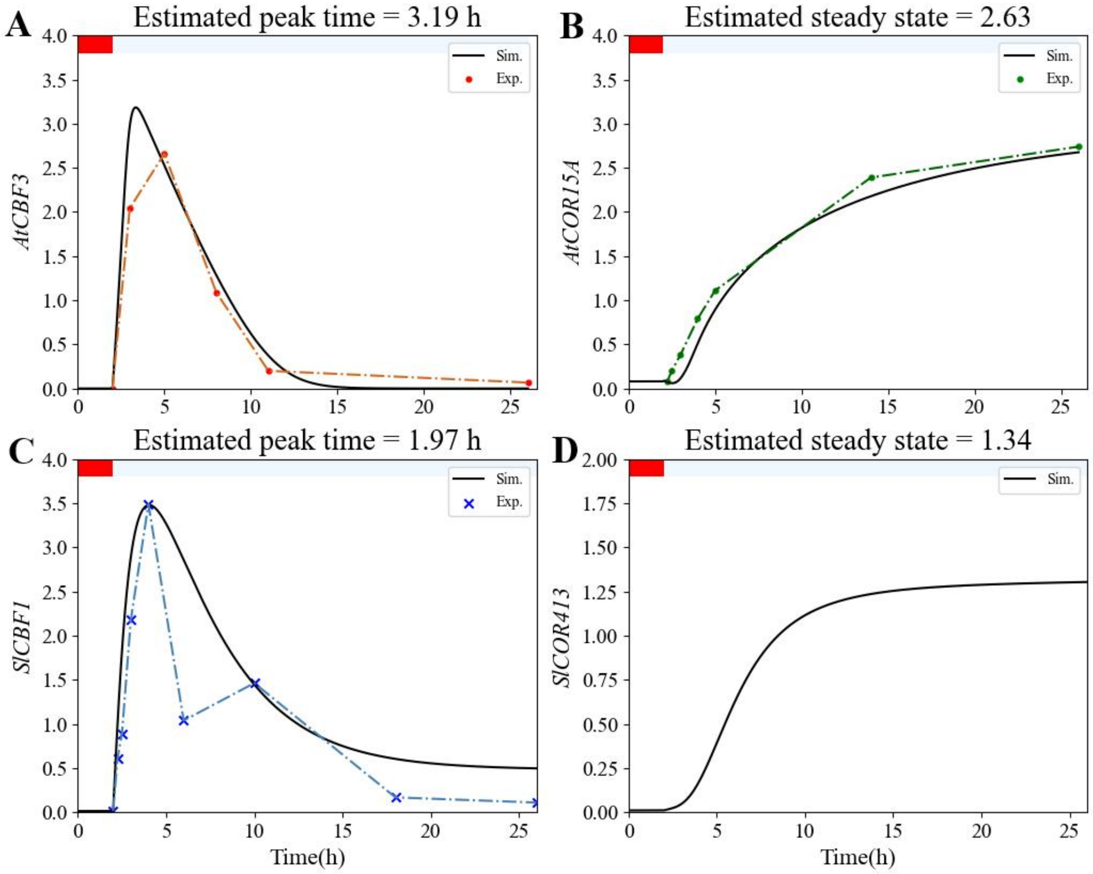

Supplementary Figure 2. Temporal dynamics of simulated versus experimental data under different plant stress models.

Panels (A) and (B) represent the *Arabidopsis* H2025 model, comparing simulation outputs with experimental observations. Panels (C) and (D) represent the tomato cold acclimation model, comparing simulation outputs with experimental observations.
