# Supplementary material for: Integrative modeling of cold and light signaling in tomato: unraveling the transcriptional network governing cold acclimation
Source: Eng Life Sci. 2026 Aug 12;26(8):e007. doi: 10.48130/els-0026-0007 (PMC13599643; doi:10.48130/els-0026-0007)
Supplement: Supplementary file 1 [file els-2026-26-8-0007-S1.zip › 10.48130_els-0026-0007-Suppl-FigureS3.pdf]

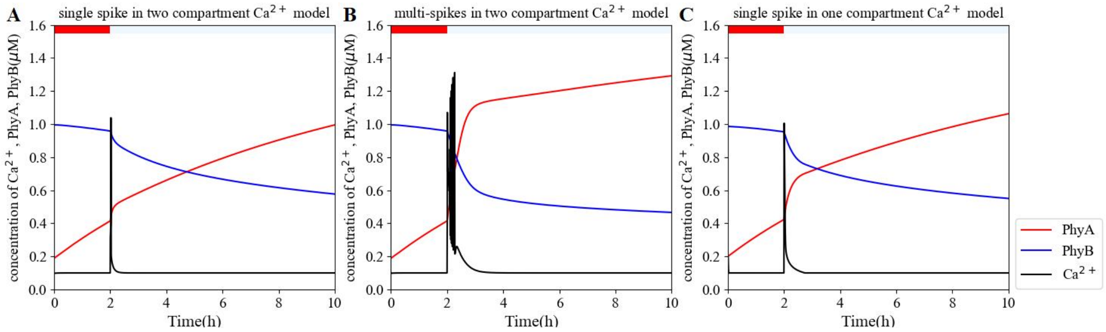

Supplementary Figure 3. Ca^{2+} signaling modes differentially shape phytochrome A/B homeostasis.

(A) Dual- compartment Ca^{2+} model under single- pulse induction. A single Ca^{2+} pulse induces gradual phyA accumulation (red curve) toward a high steady- state level, while phyB (blue curve) declines rapidly and stabilizes at a low level, establishing a "high phyA / low phyB" equilibrium. (B) Dual- compartment Ca^{2+} model under multi- pulse induction. Repetitive Ca^{2+} pulses trigger pronounced phyA and phyB fluctuations. phyA rises transiently before declining to a stable plateau, whereas phyB shows a brief increase followed by sustained decrease, indicating accelerated pigment- homeostasis establishment under oscillatory signaling. (C) Single- compartment Ca^{2+} model under single- pulse induction. Although phyA and phyB trends resemble those in the dual- compartment model, phyA accumulation is slower and phyB decline less pronounced, demonstrating that Ca^{2+} compartmentalization enhances the amplitude and kinetics of phytochrome responses.
