# Supplementary material for: Integrative modeling of cold and light signaling in tomato: unraveling the transcriptional network governing cold acclimation
Source: Eng Life Sci. 2026 Aug 12;26(8):e007. doi: 10.48130/els-0026-0007 (PMC13599643; doi:10.48130/els-0026-0007)
Supplement: Supplementary file 1 [file els-2026-26-8-0007-S1.zip › 10.48130_els-0026-0007-Suppl-TableS1.pdf]

Supplementary Table 1. Variables of the model for cold-light response pathway in tomato

Gene ID	Gene names	Variables	Definition
Solyc12g005950.2	*CONSTITUTIVE PHOTOMORPHOGENIC 1*	[SlCOP1]	COP1 protein
Solyc08g061130.3	*ELONGATED HYPOCOTYL 5*	[MSlHY5]	*SlHY5* mRNA
		[SlHY5]	HY5 protein
Solyc03g005570.3	*MYB DOMAIN PROTEIN 15*	[MSlMYB15]	*SlMYB15* mRNA
		[SlMYB15]	Unphosphorylated SlMYB15 protein
		[SlMYB15P]	Phosphorylated SlMYB15 protein
Solyc03g118310.4	*INDUCER OF CBF EXPRESSION 1*	[SlICE1]	Unphosphorylated SlICE1 protein
		[SlICE1P]	Phosphorylated SlICE1 protein
Solyc01g105230.3	*CALMODULIN-BINDING TRANSCRIPTION ACTIVATOR*	[MSlCAMTA]	*SlCAMTA* mRNA
		[SlCAMTA]	Activated SlCAMTA protein
Solyc10g044670.2	PHYTOCHROME A	[PHYA]	Phytochrome A
Solyc05g053410.3	PHYTOCHROME B	[PHYB]	Phytochrome B
Solyc07g043580.3	PHYTOCHROME INTERACTING FACTOR 4	[SlPIF4]	SlPIF4 protein
Solyc11g011260.1	GA-INSENSITIVE 4	[SlGAI4]	SlGAI4 protein
Solyc03g026270.2	*C-REPEAT BINDING FACTOR 1*	[MSlCBF1]	*SlCBF1* mRNA
		[SlCBF1]	Unphosphorylated SlCBF1 protein
		[SlCBF1P]	Phosphorylated SlCBF1 protein
Solyc03g093870.1	*ZINC FINGER PROTEIN 12*	[MSlZAT12]	*SlZAT12* mRNA
		[SlZAT12]	SlZAT12 protein
Solyc02g071210.3	*COLD REGULATED 413*	[MSlCOR413]	*SlCOR413* mRNA
		[SlCOR413]	SlCOR413 protein
