# Supplementary material for: Integrative modeling of cold and light signaling in tomato: unraveling the transcriptional network governing cold acclimation
Source: Eng Life Sci. 2026 Aug 12;26(8):e007. doi: 10.48130/els-0026-0007 (PMC13599643; doi:10.48130/els-0026-0007)
Supplement: Supplementary file 1 [file els-2026-26-8-0007-S1.zip › 10.48130_els-0026-0007-Suppl-TableS2.pdf]

Supplementary Table 2. Basic parameter values determined by long day (12L12D) and cold entrainment and parameter comparison between tomato model with Arabidopsis model (H2025).

Parameter	Description	Value in tomato model	Value in **Arabidopsis model (H2025)**
$\boldsymbol{v_1}$	synthesis of *SlHY5* mRNA	1.7 nM h^{-1}	NA
$\boldsymbol{v_2}$	synthesis of *SlMYB15* mRNA	10.6 nM h^{-1}	NA
$\boldsymbol{v_3}$	synthesis of *SlCAMTA* mRNA	5 nM h^{-1}	NA
$\boldsymbol{v_4}$	synthesis of *SlCBF1* mRNA	15.5 nM h^{-1}	34 nM h^{-1}
$\boldsymbol{v_{5a}}$	basic synthesis of *SlZAT12* mRNA	2.23 nM h^{-1}	NA
$\boldsymbol{v_5}$	synthesis of *SlZAT12* mRNA	1.25 nM h^{-1}	9.5 nM h^{-1}
$\boldsymbol{v_6}$	synthesis of *SlCOR413* mRNA	2.2 nM h^{-1}	1 nM h^{-1}
$\boldsymbol{v_{P1}}$	Maximum rate of phosphorylation of SlMYB15 protein	0.18 nM h^{-1}	NA
$\boldsymbol{v_{P2}}$	Maximum rate of dephosphorylation of SlMYB15 protein	0.45 nM h^{-1}	4 nM h^{-1}
$\boldsymbol{v_{P3}}$	Maximum rate of phosphorylation of SlICE1 protein	1.2 nM h^{-1}	NA
$\boldsymbol{v_{P4}}$	Maximum rate of dephosphorylation of SlICE1 protein	0.9 nM h^{-1}	1.2 nM h^{-1}
$\boldsymbol{v_{P5}}$	Maximum rate of phosphorylation of SlCBF1 protein	3 nM h^{-1}	2 nM h^{-1}
$\boldsymbol{v_{P6}}$	Maximum rate of dephosphorylation of SlCBF1 protein	1 nM h^{-1}	2 nM h^{-1}
$\boldsymbol{p_0}$	Apparent first-order rate constant for synthesis of SlCOP1 protein	1.5 nM h^{-1}	NA
$\boldsymbol{p_{0D}}$	Apparent first-order rate constant for synthesis of SlCOP1 protein induced by dark	0.1 nM h^{-1}	NA
$\boldsymbol{p}$	Apparent first-order rate constant for synthesis of SlICE1 protein	2 nM h^{-1}	0.18 nM h^{-1}
$\boldsymbol{p_{PIF}}$	Apparent first-order rate constant for synthesis of SlPIF4 protein	5.5 nM h^{-1}	NA
$\boldsymbol{p_{GAI}}$	Apparent first-order rate constant for synthesis of SlGAI4 protein	2.13 nM h^{-1}	NA
$\boldsymbol{p_1}$	Apparent first-order rate constant for translation of SlHY5 protein	0.3 h^{-1}	NA
$\boldsymbol{p_2}$	Apparent first-order rate constant for translation of SlMYB15 protein	0.4 h^{-1}	NA
$\boldsymbol{p_3}$	Apparent first-order rate constant for translation of SlCAMTA protein	0.8 h^{-1}	NA
$\boldsymbol{p_4}$	Apparent first-order rate constant for translation of SlCBF1 protein	5.5 h^{-1}	0.25 h^{-1}
$\boldsymbol{p_5}$	Apparent first-order rate constant for translation of SlZAT12 protein	0.66 h^{-1}	2.23 h^{-1}
$\boldsymbol{p_6}$	Apparent first-order rate constant for translation of SlCOR413 protein	1 h^{-1}	1.2 h^{-1}
$\boldsymbol{k_1}$	Maximum rate of enzymatic degradation of *SlHY5* mRNA	1.595 h^{-1}	NA
$\boldsymbol{k_2}$	Maximum rate of enzymatic degradation of *SlMYB15* mRNA	0.3 h^{-1}	NA
$\boldsymbol{k_3}$	Maximum rate of enzymatic degradation of *SlCAMTA* mRNA	2 h^{-1}	NA
$\boldsymbol{k_4}$	Maximum rate of enzymatic degradation of *SlCBF1* mRNA	1.8 h^{-1} (linear degradation)	0.55 (cold)/ 5.5 (warm) nM h^{-1} (nonlinear degradation)

$\boldsymbol{k_5}$	Maximum rate of enzymatic degradation of *SlZAT12* mRNA	$1.5\ h^{-1}$ (linear degradation)	$0.1\ nM\ h^{-1}$ (nonlinear degradation)
$\boldsymbol{k_6}$	Maximum rate of enzymatic degradation of *SlCOR413* mRNA	$1.3\ h^{-1}$ (linear degradation)	$1.25\ nM\ h^{-1}$ (nonlinear degradation)
$\boldsymbol{k_A}$	Apparent first-order rate constant for activation of PHYA by calmodulin	$2.5\ h^{-1}$	NA
$\boldsymbol{k_B}$	Apparent first-order rate constant for activation of PHYB by calmodulin	$1\ h^{-1}$	NA
$\boldsymbol{k_{BA}}$	Apparent first-order rate constant for inhibition of PHYA by PHYB	$0.4\ h^{-1}$	NA
$\boldsymbol{d}$	First-order rate constant for degradation of SlICE1 protein	$3.8\ h^{-1}$ (linear degradation)	$0.1\ nM\ h^{-1}$ (nonlinear degradation)
$\boldsymbol{d_0}$	First-order rate constant for degradation of SlCOP1 protein	$1.2\ h^{-1}$	NA
$\boldsymbol{d_{0L}}$	First-order rate constant for degradation of SlCOP1 protein induced by light	$0.5\ h^{-1}$	NA
$\boldsymbol{d_1}$	First-order rate constant for degradation of SlHY5 protein	$0.8\ h^{-1}$	NA
$\boldsymbol{d_{1L}}$	First-order rate constant for degradation of SlHY5 protein induced by light	$0.62\ h^{-1}$	NA
$\boldsymbol{d_2}$	First-order rate constant for degradation of SlMYB15 protein	$1\ h^{-1}$ (linear degradation)	$4.92\ nM\ h^{-1}$ (nonlinear degradation)
$\boldsymbol{d_3}$	First-order rate constant for degradation of SlCAMTA protein	$0.2\ h^{-1}$	NA
$\boldsymbol{d_4}$	First-order rate constant for degradation of SlCBF1 protein	$0.001\ h^{-1}$ (linear degradation)	$1.2\ nM\ h^{-1}$ (nonlinear degradation)
$\boldsymbol{d_5}$	First-order rate constant for degradation of SlZAT12 protein	$0.38\ h^{-1}$ (linear degradation)	$1.3\ nM\ h^{-1}$ (nonlinear degradation)
$\boldsymbol{d_6}$	First-order rate constant for degradation of SlCOR413 protein	$0.2\ h^{-1}$ (linear degradation)	$0.3\ nM\ h^{-1}$ (nonlinear degradation)
$\boldsymbol{d_A}$	First-order rate constant for degradation of SlPHYA protein	$0.534\ h^{-1}$	NA
$\boldsymbol{d_B}$	First-order rate constant for degradation of SlPHYB protein	$0.954\ h^{-1}$	NA
$\boldsymbol{d_{PIF}}$	First-order rate constant for degradation of SlPIF4 protein	$1\ h^{-1}$	NA
$\boldsymbol{d_{GAI}}$	First-order rate constant for degradation of SlGAI4 protein	$0.5\ h^{-1}$	NA
$\boldsymbol{d_{P2}}$	First-order rate constant for degradation of phosphorylated SlMYB15 protein	$1\ h^{-1}$	NA
$\boldsymbol{d_{P3}}$	First-order rate constant for degradation of phosphorylated SlICE1 protein	$0.5\ h^{-1}$	NA
$\boldsymbol{d_{P4}}$	First-order rate constant for degradation of phosphorylated SlCBF1 protein	$0.5\ h^{-1}$ (linear degradation)	$1.2\ nM\ h^{-1}$ (nonlinear degradation)
$\boldsymbol{K_1}$	Threshold constant for inhibition by SlCOP1 of *SlHY5* expression	0.65 nM	NA
$\boldsymbol{K_2}$	Threshold constant for activation by SlHY5 of *SlMYB15* expression	3.5 nM	NA
$\boldsymbol{K_3}$	Threshold constant for activation by SlICE1 of *SlCBF1* expression	0.1 nM	0.21
$\boldsymbol{K_4}$	Threshold constant for activation by SlMYB15 of *SlCBF1* expression	0.8 nM	0.8 nM

$\boldsymbol{K_5}$	Threshold constant for activation by SlHY5 of *SlCBF1* expression	0.01 nM	NA
$\boldsymbol{K_6}$	Threshold constant for activation by SlPIF4 of *SlCBF1* expression	0.6 nM	NA
$\boldsymbol{K_7}$	Threshold constant for activation by SlCAMTA of *SlCBF1* expression	0.3 nM	NA
$\boldsymbol{K_8}$	Threshold constant for inhibition by SlZAT12 of *SlCBF1* expression	0.52 nM	0.3 nM
$\boldsymbol{K_9}$	Threshold constant for activation by SlCBF1 of *SlZAT12* expression	50 nM	0.28 nM
$\boldsymbol{K_{10}}$	Threshold constant for activation by SlCBF1 of *SlCOR413* expression	80 nM	0.1 nM
$\boldsymbol{K_{11}}$	Threshold constant for activation by SlZAT12 of *SlCOR413* expression	3.6 nM	0.2 nM
$\boldsymbol{K_{P1}}$	Michaelis constant for phosphorylation of SlMYB15 protein	0.6 nM	0.5 nM
$\boldsymbol{K_{P2}}$	Michaelis constant for dephosphorylation of SlMYB15 protein	0.5 nM	0.5 nM
$\boldsymbol{K_{P3}}$	Michaelis constant for phosphorylation of SlICE1 protein	0.05 nM	0.45 nM
$\boldsymbol{K_{P4}}$	Michaelis constant for dephosphorylation of SlICE1 protein	1.2 nM	0.8 nM
$\boldsymbol{K_{P5}}$	Michaelis constant for phosphorylation of SlCBF1 protein	0.6 nM	0.6 nM
$\boldsymbol{K_{P6}}$	Michaelis constant for dephosphorylation of SlCBF1 protein	0.58 nM	0.58 nM
$\boldsymbol{K_{CaM1}}$	Michaelis constant for phosphorylation of SlMYB15 protein induced by calmodulin	3 nM	0.5 nM
$\boldsymbol{K_{CaM2}}$	Michaelis constant for phosphorylation of SlICE1 protein induced by calmodulin	0.45 nM	0.45 nM
$\boldsymbol{K_{CaM3}}$	Threshold constant for activation of *SlCAMTA* expression induced by calmodulin	1.5 nM	NA
$\boldsymbol{K_A}$	Michaelis constant for phosphorylation of SlPHYA protein induced by calmodulin	1 nM	NA
$\boldsymbol{K_B}$	Michaelis constant for phosphorylation of SlPHYB protein induced by calmodulin	0.1 nM	NA
$\boldsymbol{K_{AB}}$	Threshold constant for inhibition of SlPHYA expression by SlPHYB	1.5 nM	NA
$\boldsymbol{K_{BA}}$	Threshold constant for inhibition of SlPHYB expression by SlPHYA	0.23 nM	NA
$\boldsymbol{K_{phyA}}$	Threshold constant for inhibition of SlPIF4 expression by SlPHYA	1 nM	NA
$\boldsymbol{K_{phyB}}$	Threshold constant for inhibition of SlPIF4 expression by SlPHYB	0.6 nM	NA
$\boldsymbol{K_{PIF}}$	Threshold constant for activation of SlGAI4 expression by SlPIF4	0.5 nM	NA
$\boldsymbol{K_{GAI}}$	Threshold constant for inhibition of SlPIF4 expression by SlGAI4	0.2 nM	NA
$\boldsymbol{\alpha_{FR}}$	Michaelis constant for degradation of PHYA under low R:FR	0.2 nM	NA
$\boldsymbol{\alpha_R}$	Michaelis constant for degradation of PHYB under high R:FR	0.4 nM	NA

Note: The degradation rate units differ between the two models because linear degradation is applied in the Tomato model, whereas nonlinear degradation is used in H2025.
