# Supplementary material for: Integrative modeling of cold and light signaling in tomato: unraveling the transcriptional network governing cold acclimation
Source: Eng Life Sci. 2026 Aug 12;26(8):e007. doi: 10.48130/els-0026-0007 (PMC13599643; doi:10.48130/els-0026-0007)
Supplement: Supplementary file 1 [file els-2026-26-8-0007-S1.zip › 10.48130_els-0026-0007-Suppl-TableS3.pdf]

Supplementary Table 3. Goodness-of-fit indicators between experimental expressions and model simulations.

Target gene	Dataset	R^2	RMSE
SlHY5	Training (Zhang et al. 2020)	0.8965	0.1654
SlMYB15	Training (Zhang et al. 2020)	0.8362(WT)/0.9175(mutant)/0.885(OE)	0.23506/0.1301/0.36965
SlCBF1	Validation (Zhang et al. 2004)	0.8434/0.9859	0.58285/0.1937
SlCOR413	Validation (Zhang et al. 2021)	0.9283	0.09034
