# Supplementary material for: Integrative modeling of cold and light signaling in tomato: unraveling the transcriptional network governing cold acclimation
Source: Eng Life Sci. 2026 Aug 12;26(8):e007. doi: 10.48130/els-0026-0007 (PMC13599643; doi:10.48130/els-0026-0007)
Supplement: Supplementary file 1 [file els-2026-26-8-0007-S1.zip › 10.48130_els-0026-0007-Suppl-text1.pdf]

Supplementary Text 1. Construction of the integrated light and cold signaling network.

1.1 The core cold response pathway: ICE1-CBF-COR

The core module comprises the MYC-type bHLH transcription factor INDUCER OF CBF EXPRESSION 1 (ICE1), the C-repeat binding factor CBF1, and the cold-regulated gene *COR413*. ICE1 functions as an early activator of cold responses and binds to the *CBF3* promoter. Cold-induced post-translational modifications, particularly phosphorylation, are required for ICE1 to activate downstream genes. In the model, a calmodulin (CaM)-activated kinase negatively regulates SlICE1 by promoting its phosphorylation under cold conditions [1]. Expression of SlCBF1 is activated by multiple transcription factors including SlCAMTA, SlHY5, SlMYB15, SlICE1, and SlPIF4, and is inhibited by SlZAT12 [2–6]. Phosphorylation of SlCBF1 is modulated by a CaM-dependent kinase [7]. Downstream, SlCBF1 together with SlZAT10 activates SlCOR413 transcription [8], leading to enhanced cold acclimation. Two auxiliary regulatory components are incorporated into this module. First, calcium signaling via calmodulin drives SlCAMTA expression, and CAMTA proteins positively regulate CBF genes in a Ca^{2+} /calmodulin-dependent manner [9, 10]. Second, the zinc finger proteins ZAT10 and ZAT12 modulate the CBF pathway through a negative feedback loop: elevated ZAT12 represses CBF expression, while CBF3 activates ZAT12 [11].

1.2 The light signal module: COP1-HY5-MYB15

The second module mediates the regulation of cold responses by light and centers on the E3 ubiquitin ligase CONSTITUTIVE PHOTOMORPHOGENIC 1 (COP1), the bZIP transcription factor ELONGATED HYPOCOTYL 5 (HY5), and the MYB transcription factor MYB15. Light suppresses COP1 synthesis [12]. In darkness, COP1 protein represses HY5 transcription and promotes HY5 degradation. Under light conditions, COP1 is inhibited, leading to HY5 stabilization and accumulation [13, 14]. HY5 directly binds to G-box and ACE-box promoter elements to activate

downstream target genes, including MYB15 [6, 15, 16]. Low temperature further enhances HY5 protein stability [15]. In tomato, SlMYB15 functions as a transcriptional activator of cold signaling: SlHY5 directly binds the SlMYB15 promoter to enhance its transcription, and SlMYB15 subsequently activates CBF genes [6].

1.3 The light quality signal module: phytochromes-PIF4-GAI4

The third module responds to changes in light quality (red to far-red ratio) and integrates light signals with temperature cues. Its core components include the phytochrome photoreceptors phyA and phyB, the bHLH transcription factor PHYTOCHROME INTERACTING FACTOR 4 (PIF4), and the DELLA protein GIBBERELLIC ACID INSENSITIVE 4 (GAI4). PhyA perceives far-red (FR) light while phyB perceives red (R) light, and the two photoreceptors exhibit mutual inhibition. They modulate stress responses via abscisic acid- and jasmonic acid-dependent pathways [17, 18]. Under cold stress, low R:FR light and low temperature promote SlPIF4 protein accumulation through a phyA-dependent pathway, whereas low temperature restricts SlPIF4 accumulation via phyB [19]. SlPIF4 directly activates SlGAI4 transcription, and SlGAI4 protein in turn inhibits SlPIF4 accumulation, forming a coherent feed-forward loop that fine-tunes cold responses [5]. Because SlGAI4 interacts only with SlPIF4 within the model framework, its mRNA and protein were combined into a single variable.

1.4 Integration of the three modules

The three modules are coupled into a complete signal transduction network through shared transcription factors and common environmental inputs. The COP1-HY5-MYB15 module inputs light signals into the core cold response pathway via HY5- and MYB15-mediated activation of CBF1 expression. The phytochromes-PIF4-GAI4 module integrates light quality and temperature signals and inputs them into the core pathway via PIF4-mediated activation of CBF1. The ICE1-CBF-COR module serves as the common downstream output pathway, receiving activating inputs from HY5, MYB15, PIF4, CAMTA, and ICE1, and an inhibitory input from ZAT12.

Additionally, CaM signaling forms a calcium-coupled layer that simultaneously regulates CAMTA expression, ICE1 phosphorylation, and CBF1 phosphorylation. To maintain clarity while retaining biological relevance, certain simplifications were made: kinases activated by elevated cytosolic Ca^{2+} were represented as a unified module, and only degradation pathways explicitly regulated by light or low temperature are depicted in detail. All other processes follow mass-action or Michaelis- Menten kinetics as described in the Supplementary Materials.

1.5 Mathematical model formulation

Based on the integrated network described above, a mathematical model was constructed as a system of 24 ordinary differential equations (ODEs). Each equation describes the rate of change of a molecular species (mRNA or protein) and includes terms for synthesis, degradation, activation, inhibition, and phosphorylation. Kinetic parameters were derived from or calibrated to experimental data from tomato and, where homologous information was available, from Arabidopsis. Environmental inputs to the model include light (presence versus absence, intensity, and red to far-red ratio) and temperature (normal versus cold stress). The model captures the following key regulatory events: light suppression of COP1 synthesis; light stabilization of HY5 protein; low-temperature enhancement of HY5 stability and MYB15 phosphorylation; antagonistic regulation of PIF4 accumulation by phyA and phyB under low R:FR light and low temperature; CaM-mediated regulation of CAMTA, ICE1, and CBF1 phosphorylation; cooperative activation of CBF1 by five transcription factors (CAMTA, HY5, MYB15, ICE1, and PIF4); feedback repression of CBF1 by ZAT12; and feedback repression of PIF4 by GAI4.

References

[1] Dong, C.H., Agarwal, M., Zhang, Y., Xie, Q., et al., The negative regulator of plant cold responses, HOS1, is a RING E3 ligase that mediates the ubiquitination

and degradation of ICE1. *Proceedings of the National Academy of Sciences* 2006, *103*, 8281–8286.

[2] Yuasa, T., Tomato Inducer of CBF Expression 1 (SlICE1) is involved in cold and salt stress signaling. *AJEA* 2014, *4*, 785–796.

[3] Chen, Y., Lv, C., Li, F., Li, T., Distinguishing the rates of gene activation from phenotypic variations. *BMC Syst Biol* 2015, *9*, 29.

[4] Iqbal, Z., Shariq Iqbal, M., Singh, S.P., Buaboocha, T., Ca2+/Calmodulin complex triggers CAMTA transcriptional tachinery under stress in plants: signaling cascade and molecular regulation. *Front. Plant Sci.* 2020, *11*, 598327.

[5] Wang, M., Hao, J., Chen, X., Zhang, X., SlMYB102 expression enhances low-temperature stress resistance in tomato plants. *PeerJ* 2020, *8*, e10059.

[6] Zhang, L., Jiang, X., Liu, Q., Ahammed, G.J., et al., The HY5 and MYB15 transcription factors positively regulate cold tolerance in tomato via the CBF pathway. *Plant Cell Environ* 2020, *43*, 2712–2726.

[7] Liu, Z., Jia, Y., Ding, Y., Shi, Y., et al., Plasma membrane CRPK1-mediated phosphorylation of 14-3-3 proteins induces their nuclear import to fine-tune CBF signaling during cold response. *Molecular Cell* 2017, *66*, 117-128.e5.

[8] Zhang, L., Guo, X., Zhang, Z., Wang, A., et al., Cold-regulated gene LeCOR413PM2 confers cold stress tolerance in tomato plants. *Gene* 2021, *764*, 145097.

[9] Doherty, C.J., Van Buskirk, H.A., Myers, S.J., Thomashow, M.F., Roles for *Arabidopsis* CAMTA transcription factors in cold-regulated gene expression and freezing tolerance. *The Plant Cell* 2009, *21*, 972–984.

[10] Park, S., Gilmour, S.J., Grumet, R., Thomashow, M.F., CBF-dependent and CBF-independent regulatory pathways contribute to the differences in freezing tolerance and cold-regulated gene expression of two Arabidopsis ecotypes locally adapted to sites in Sweden and Italy. *PLoS ONE* 2018, *13*, e0207723.

[11] Zhang, R., Gonze, D., Hou, X., You, X., et al., A computational model for the cold response pathway in plants. *Frontiers in Physiology* 2020, *11*, 591073.

[12] Lau, O.S., Deng, X.W., Plant hormone signaling lightens up: integrators of light and hormones. *Curr. Opin. Plant Biol.* 2010, *13*, 571–577.

[13] Gangappa, S.N., Botto, J.F., The multifaceted roles of HY5 in plant growth and development. *Molecular Plant* 2016, *9*, 1353–1365.

[14] Burko, Y., Seluzicki, A., Zander, M., Pedmale, U.V., et al., Chimeric activators and repressors define HY5 activity and reveal a light-regulated feedback mechanism. *Plant Cell* 2020, *32*, 967–983.

[15] Catala, R., Medina, J., Salinas, J., Integration of low temperature and light signaling during cold acclimation response in Arabidopsis. *Proceedings of the National Academy of Sciences* 2011, *108*, 16475–16480.

[16] Shin, D.H., Choi, M., Kim, K., Bang, G., et al., HY5 regulates anthocyanin biosynthesis by inducing the transcriptional activation of the MYB75/PAP1 transcription factor in *Arabidopsis*. *FEBS Letters* 2013, *587*, 1543–1547.

[17] Wang, F., Guo, Z., Li, H., Wang, M., et al., Phytochrome A and B function antagonistically to regulate cold tolerance via abscisic acid-dependent Jasmonate signaling. *Plant Physiol.* 2016, *170*, 459–471.

[18] Yang, F., Feng, L., Liu, Q., Wu, X., et al., Effect of interactions between light intensity and red-to- far-red ratio on the photosynthesis of soybean leaves under shade condition. *Environ. Exp. Bot.* 2018, *150*, 79–87.

[19] Wang, X., Gao, X., Liu, Y., Fan, S., et al., Progress of research on the regulatory Pathway of the plant shade-avoidance syndrome. *Front. Plant Sci.* 2020, *11*, 439.
