# Supplementary material for: Integrative modeling of cold and light signaling in tomato: unraveling the transcriptional network governing cold acclimation
Source: Eng Life Sci. 2026 Aug 12;26(8):e007. doi: 10.48130/els-0026-0007 (PMC13599643; doi:10.48130/els-0026-0007)
Supplement: Supplementary file 1 [file els-2026-26-8-0007-S1.zip › 10.48130_els-0026-0007-Suppl-text2.pdf]

Supplementary Text 2. Period and phase calculation.

To characterize the oscillatory behavior of gene expression, time-series data for core circadian clock genes and photosynthetic genes were collected from both experimental measurements and model simulations. For each oscillation, we identified key features including the peak (maximum), trough (minimum), and the midpoint between them, which represents the baseline crossing point. These features were located using the find_peak function from the scipy.signal module in Python. A reference time point was then chosen, based on the external light–dark cycle and defined as Zeitgeber Time (ZT). The phase of a given feature, such as the peak, was calculated as the time difference (in hours) between the occurrence of that feature and the reference time: phase = time_of_feature − reference_time. Finally, the period of the oscillation was determined by measuring the interval between two consecutive peaks or two consecutive troughs.
