# Supplementary material for: Integrative modeling of cold and light signaling in tomato: unraveling the transcriptional network governing cold acclimation
Source: Eng Life Sci. 2026 Aug 12;26(8):e007. doi: 10.48130/els-0026-0007 (PMC13599643; doi:10.48130/els-0026-0007)
Supplement: Supplementary file 1 [file els-2026-26-8-0007-S1.zip › 10.48130_els-0026-0007-Suppl-text3.pdf]

Supplementary Text 3. Parameter estimation.

This study's parameter estimation is based on published experimental data from tomato [6, 20], specifically the relative expression profiles of core genes under combined light and temperature signals (Figure 3A). The fitting process is defined as follows: under a low-temperature stress condition (4°C) and a 12-hour light/12-hour dark photoperiod (12L:12D), we used the relative expression data of the *SlHY5* gene in wild-type plants as the primary fitting target. Simultaneously, the expression data of *SlMYB15* in wild-type, *hy5* mutant, and HY5-overexpression plants under identical conditions were integrated to establish correlations between gene expression and model parameters.

Model parameters were iteratively optimized using a least-squares error method to minimize the deviation between simulated and experimentally measured expression values for *SlHY5* and *SlMYB15*, yielding an optimal parameter set.

To validate the optimized parameters and the model's predictive capability, we simulated the relative expression of downstream genes *SlCBF1* and *SlCOR413* under altered light conditions (24L and 16L:8D). Comparing these simulations with potential experimental patterns verified the model's stability and applicability, providing a reliable foundation for analyzing the molecular mechanisms of light-temperature signal integration in tomato cold acclimation.

References

[6] Zhang, L., Jiang, X., Liu, Q., Ahammed, G.J., et al., The HY5 and MYB15 transcription factors positively regulate cold tolerance in tomato via the CBF pathway. *Plant Cell Environ* 2020, *43*, 2712–2726.

[20] Han, N., Fan, S., Zhang, T., Sun, H., et al., SlHY5 is a necessary regulator of the cold acclimation response in tomato. *Plant Growth Regul* 2020, *91*, 1–12.
